# Sound Insulation of Corrugated-Core Sandwich Panels: Modeling, Optimization and Experiment

**DOI:** 10.3390/ma14247785

**Published:** 2021-12-16

**Authors:** Longlong Ren, Haosen Yang, Lei Liu, Chuanlong Zhai, Yuepeng Song

**Affiliations:** 1 College of Mechanical and Electronic Engineering, Shandong Agricultural University, Tai’an 271018, China; renlonglong@sdau.edu.cn (L.R.); euliulei@hotmail.com (L.L.); zchuanlong@hotmail.com (C.Z.); 2 Shandong Provincial Engineering Laboratory of Agricultural Equipment Intelligence, Tai’an 271018, China; 3 Shandong Key Laboratory of Garden Machinery and Equipment, Tai’an 271018, China; 4 Chongqing Bashu Secondary School, Chongqing 400013, China; haosenyang@hotmail.com

**Keywords:** sandwich panel, corrugated core, vibro-acoustic optimization, coefficient of determination, sound insulation

## Abstract

With the extension of the applications of sandwich panels with corrugated core, sound insulation performance has been a great concern for acoustic comfort design in many industrial fields. This paper presents a numerical and experimental study on the vibro-acoustic optimization of a finite size sandwich panel with corrugated core for maximizing the sound transmission loss. The numerical model is established by using the wave-based method, which shows a great improvement in the computational efficiency comparing to the finite element method. Constrained by the fundamental frequency and total mass, the optimization is performed by using a genetic algorithm in three different frequency bands. According to the optimization results, the frequency averaged sound transmission of the optimized models in the low, middle, and high-frequency ranges has increased, respectively, by 7.6 dB, 7.9 dB, and 11.7 dB compared to the baseline model. Benefiting from the vast number of the evolution samples, the correlation between the structural design parameters and the sound transmission characteristics is analyzed by introducing the coefficient of determination, which gives the variation of the importance of each design parameter in different frequency ranges. Finally, for validation purposes, a sound insulation test is conducted to validate the optimization results in the high-frequency range, which proves the feasibility of the optimization method in the practical engineering design of the sandwich panel.

## 1. Introduction

Because of its high stiffness to mass ratio and excellent impact resistance property, lightweight sandwich panels are largely used in civil construction, high-speed vehicle, ship structure, and aerospace industries [1,2]. A typical sandwich panel usually consists of two face sheets and a core layer. According to the different types of the core, sandwich panels can be broadly separated into two categories, homogeneous core sandwich panels and non-homogeneous (structure supported) core sandwich panels. Due to the lack of mechanical strength, the former type (e.g., foam core sandwich panel) is commonly used in the applications under light load condition. To overcome this drawback, the latter type, non-homogeneous sandwich panel, uses structural stiffener as the core layer. The core structure not only provides additional mechanical strength and bending stiffness but can also keep the lightweight characteristics of the sandwich panel. With the extension of the applications of sandwich panels, the vibro-acoustic properties, especially the sound insulation performance, have attracted great attention for acoustic comfort design in many industrial fields [3].

Due to the geometry diversity of the structural core, the mechanism of sound transmission through structure supported sandwich plate is complicated. Under this circumstance, many theoretical models were proposed to study the basic vibro-acoustic behaviors of structure supported sandwich plate. In the early stage, beginning with investigating sound transmission through building element, Sharp [4] studied the sound insulation performances of structure reinforced double wall structure and proposed an analytical model to predict the sound transmission loss. In this model, the structural reinforcement was assumed as totally rigid, and the link impedance was introduced to evaluate the sound transmission via structural path. Fahy [5] calculated the sound reduction index of double-leaf partitions with timber and steel studs based on the same rigid assumption. The comparison between the calculated results and experimental data indicated that this highly idealized theoretical model works well when the two face sheets are linked by inelastic studs.

As for the elastic structure linked sandwich plates, Brunskog [6] presented a deterministic prediction model for airborne sound insulation performances of an infinite double leaf structure. In his study, both the structural and acoustical (acoustic cavities) sound transmission paths are considered. However, as a simplification, the periodically placed orthogonal studs were treated as bar-like beams, and only the axial displacement was considered. Taking the flexural vibration of structural stiffeners into consideration, Xin and Lu [7] proposed an analytical model to evaluate the sound transmission of lightweight all-metallic sandwich panels. In their study, the structural stiffeners were simplified as translational and rotational springs with concentrated mass. Meanwhile, the structural–acoustical coupling effect was assumed to take place only between the acoustic cavity and the two face sheets. The results indicated that the core geometry exerts a significant effect on the sound insulation performance of the sandwich plate.

Due to the complexity of the vibro-acoustical coupling system, most of the aforementioned theoretical models can only handle the sandwich plates whose core structures are relatively simple (e.g., periodic orthogonality stiffeners). For those sandwich structure with complex core, such as honeycomb core, homogenization techniques [8] are used to reduce the structural core to an equivalent homogeneous material, and as a result, the sandwich plate can be treated as a simple layered structure [9]. However, the homogenization model can only provide approximate results due to the ignoring of physical details of the sandwich structure. To gain a full understanding of sound transmission through the sandwich plate, the conventional finite element method is preferred to perform the sound transmission analysis of the sandwich structure with complex core [10]. For instance, Ruzzene [11] studied the vibration and sound radiation properties of the sandwich structure with a honeycomb truss core with the spectral finite element method, and the sound transmission reduction indexes of sandwich beams with different core configurations were evaluated and compared. Kim and Han [12] performed an investigation on the acoustic characteristics of honeycomb sandwich composite panels by using the finite element method associated with the boundary element method.

According to the results obtained from the aforementioned analytical and numerical investigations on the sound transmission through sandwich structures, the sound transmission characteristics and the stiffness-to-weight ratio of the sandwich panels are very sensitive to the core topology and material properties. In order to improve the sound insulation properties of the sandwich structure without losing any mechanical strength, the vibro-acoustic optimization of the sandwich structures has been studied by many researchers [13,14,15].

Under the constraints of total structural mass and the first fundamental frequency, Denli and Sun [16] conducted optimization of sandwich beam with cellular cores with the aim of maximizing the sound transmission loss (STL) in different frequency bands. Because all the nodal displacements were considered as the design variables, the sensitivity functions were introduced to improve the computational time and accuracy. Despite the significant reduction of sound radiation was achieved in this study, the optimization led to cellular cores with random and nonperiodic geometry, which have very low manufacturability. Franco et al. [17] presented a structural-acoustic optimization for minimizing the structural acoustic response of sandwich panels with various core configurations (e.g., random core and truss-like core). The study of optimization used the MSC/NASTRAN optimizer (SOL 200) based on modified method of feasible directions. The optimization results indicated that the structural acoustic response of the sandwich structure could be tailored for specific applications. In this study, the finite element method was used to calculate the objective function, which is suitable for modeling complex geometries. Most recently, Thompson and Galgalikar [18] also used the finite element method with MODEFRONTIER, a general purpose optimization software, to conduct a structural–acoustic optimization targeting at maximizing the STL response of the sandwich panel with honeycomb core with in-plane orientation. Unlike the conventional optimization procedure, the geometrical parameters of a unit cell were treated as the design parameters to keep the periodicity of the core structure. In addition to obtaining a significant increase of STL, the optimization results indicated that the acoustic response of the sandwich panel strongly depends on the number of unit cells in the horizontal and vertical direction.

Despite the fact that the detailed structural–acoustic response of the sandwich structure can be determined numerically, e.g., through the use of the finite element method (FEM) or the boundary element method (BEM), computational efficacy is still a drawback [19]. For the optimization procedure, which usually contains massive loop computations, a more efficient alternative method is needed.

As a derivation of the indirect Trefftz approach [20], the wave-based method (WBM) was first proposed by Desmet [21] in the 90s. It differs from the element-based methods (e.g., FEM) in that, instead of discretizing the physical field into a large number of tiny elements, the WBM divides the targeting problem domain into a small number of subdomains. In each subdomain, the field variables are approximated by a weighted sum of a series of frequency dependent wave functions, and each wave function is the exact solution of the governing differential equations. In this respect, the WBM leads to a much smaller number of system DOFs than the conventional element-based methods [22]. Thus far, the WBM has been successfully implemented in solving the bounded acoustic problems [23], acoustic radiation and scattering problems [24], structural vibration [25], and simple acoustical–structural coupling problems [26]. As opposed to the large (geometrical) flexibility in element-based methods, the WBM is limited to only a limited number of (convex) subdomains to remain computationally favorable.

In this paper, the sandwich panel with corrugated core is modeled as a vibro-acoustic coupled system, where the effect of the acoustic cavities in the core layer has also been taken into consideration. The vibro-acoustic response of the system is calculated by using the wave-based method. In association with the genetic algorithm (GA) and with the advantage of the high computational efficiency of WBM, a vibro-acoustic optimization is performed to maximize the sound transmission loss of the sandwich structure.

This paper is organized as follows. Firstly, the model configuration is presented to offer a basic understanding of the problem. Then, based on the governing equations of the vibro-acoustic system, the wave-based method is implemented to solve the vibro-acoustic response of the sandwich structure. By using the Rayleigh’s integral, the radiated sound power is obtained from the vibrating system. Secondly, the genetic algorithm is used to perform the vibro-acoustic optimization aiming at maximizing the sound transmission loss in different frequency ranges. According to the optimization history, a statistical analysis is implemented to calculate the coefficient of determination (COD) of each design parameter and reveal the strength of the relationship between each design parameter and the STL. Finally, a confirmatory sound insulation test on a real sandwich panel specimen is conducted to verify the optimization configuration.

## 2. Theoretical Formulation

### 2.1. Model Configuration

**Figure 1:**
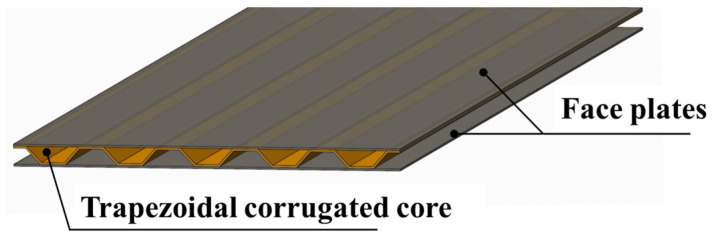
Model geometry of sandwich panel with trapezoidal corrugated core.

A typical sandwich panel usually consists of two face sheets and a structural core layer. Among various types of core structure, the corrugated core has attracted growing attention thanks to its cost and manufacturing advantages [27]. The structure is a sandwich panel with trapezoidal corrugated core, as shown in Figure 1.

Due to the existence of periodical stiffening structure, the original rectangular cavity between the two face panels is divided into several trapezoidal sub-cavities.

**Figure 2:**
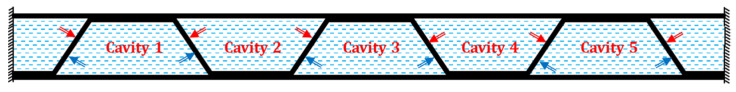
The vibro-acoustic coupling between the structure and trapezoidal acoustic cavities.

As shown in Figure 2, since the vibro-acoustic coupling effect between the internal acoustic cavities and the structure is taken into consideration, the classic mass–air–mass resonance in a simple double-leaf structure is turned into the complex vibro-acoustic coupling between each trapezoidal acoustic cavity and its surrounding structure. Thus, the incident sound wave can be transmitted to the top face sheet through the structural and acoustical borne paths and finally radiate to the semi-infinite domain.

### 2.2. Theoretical Formulation

Considering the periodical one-way stiffened feature of the corrugated core, the sandwich panel studied in this paper is assumed to have an infinite length in the reinforced direction [28]. As a result, the dynamic response of the sandwich panel can be simplified into a plane strain problem, and only the cross section of the sandwich structure is considered.

**Figure 3:**
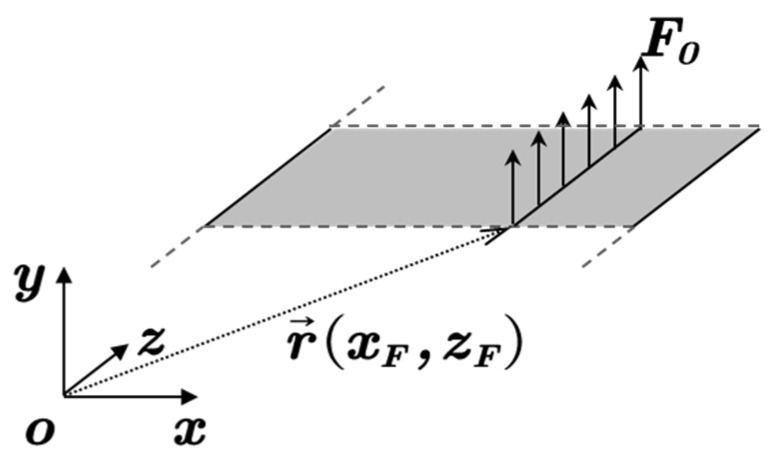
Model of a single elastic panel.

In terms of structure, the sandwich panel can be treated as an assembly of several single panel, as shown in Figure 3.

According to the Kirchhoff theory [29] and Navier equations [30], the governing equations of the free transverse and longitudinal vibration of a thin panel can be expressed as

(1) $$\left\{ \begin{array}{l} (\frac{\partial^{4}w}{\partial x^{4}}+2\frac{\partial^{4}w}{\partial x^{2}\partial y^{2}}+\frac{\partial^{4}w}{\partial y^{4}})-\frac{\rho h\omega^{2}}{D}w=0\\ \frac{\partial^{2}u}{\partial x^{2}}+\frac{1-\nu }{2}\frac{\partial^{2}u}{\partial y^{2}}+\frac{1+\nu }{2}\frac{\partial^{2}v}{\partial x\partial y}+\omega^{2}\cdot \frac{\rho (1-\nu^{2})}{E}u=0\\ \frac{\partial^{2}v}{\partial y^{2}}+\frac{1-\nu }{2}\frac{\partial^{2}v}{\partial x^{2}}+\frac{1+\nu }{2}\frac{\partial^{2}u}{\partial x\partial y}+\omega^{2}\cdot \frac{\rho (1-\nu^{2})}{E}v=0 \end{array}\right.$$

where w is the transverse displacement, and u is the longitudinal displacement.

In addition,

(2) $$D=\frac{E(1+i\eta )h^{3}}{12(1-\nu^{2})}$$

where ν is the Poisson’s ratio, and ρ and h are the material density and thickness of the panel, respectively. Associating with the elasticity modulus E, in order to consider the material damping, the damping factor η is added and leads to a complex elasticity modulus $i=\sqrt{-1}$.

**Figure 4:**
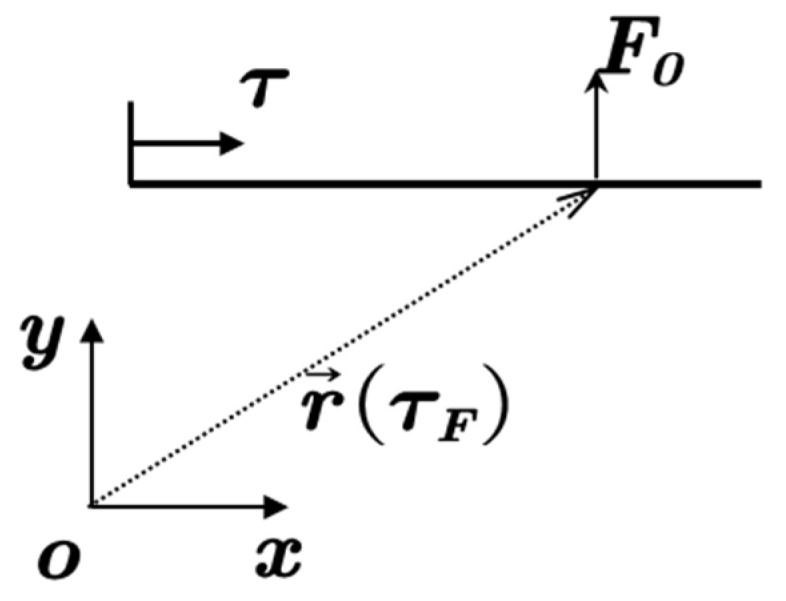
Degradation model of flexural vibration problem of elastic panel.

With the assumption of infinite length, as shown in Figure 4, Equation (1) degenerate to

(3) $$\left\{ \begin{array}{l} \frac{\partial^{4}w(\tau )}{\partial \tau^{4}}-k_{b}^{4}\cdot w(\tau )=0\\ \frac{\partial^{2}u(\tau )}{\partial \tau^{2}}+k_{l}^{2}\cdot u(\tau )=0 \end{array}\right.$$

where τ is the local coordinate of the panel, and

(4) $$k_{b}=\sqrt[{4}]{\frac{\rho h\omega^{2}}{D}},k_{l}^{2}=\omega^{2}\cdot \frac{\rho (1-\nu^{2})}{E}$$

where $k_{b}$ and $k_{l}$ are the bending and longitudinal wave number, respectively.

**Figure 5:**
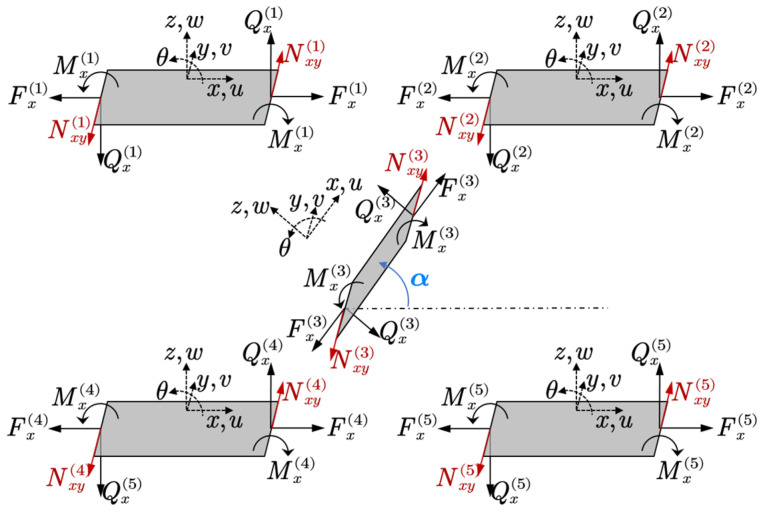
The demonstration of displacement compatibility conditions and force equilibrium conditions.

For the structural part, since the sandwich structure is an assembly of several single plates and both the transverse and longitudinal displacements are expressed by the summation of corresponding wave functions, the governing equations of the whole structure can be assembled based on the displacement compatibility conditions and force equilibrium conditions. A demonstration of the connection relations between five sub-plates is shown in Figure 5. According to the force–displacement relation, all the internal forces can be calculated by using the following relations:

(5) $$\theta =\frac{dw}{d\tau },M_{x}=D\frac{d^{2}w}{d\tau^{2}},Q_{x}=-D\frac{d^{3}w}{d\tau^{3}},F_{x}=\frac{Eh}{\left(1-\nu^{2}\right)}\frac{du}{d\tau }$$

As for the acoustic cavity, the governing equation of the sound pressure inside is the Helmholtz equation [24]:

(6) $$\nabla^{2}p_{a}(r)+k^{2}p_{a}(r)=0,k=\frac{\omega }{c}$$

where c is the sound speed inside the acoustic cavity, and k is the acoustic wave number. According to the basic concept of the WBM, both the structural displacement and sound pressure can be approximated by a summation of the specific wave functions [22].

(7) $$\left\{ \begin{array}{l} \hat{w}(r)=\sum_{n=1}^{n_{s}}\Psi_{b,n}(r)\cdot \xi_{n}+{\tilde{w}}_{a}(r)+{\tilde{w}}_{q}(r)+{\tilde{w}}_{F}(r)\\ \hat{u}(r)=\sum_{i}^{2}\Psi_{l,i}\cdot \zeta_{l,i}\\ {\hat{p}}^{\left(\alpha \right)}(r)=\sum_{m=1}^{m_{a}}\in_{m}\cdot \Phi_{m}^{\left(\alpha \right)}(r)+{\hat{p}}_{q}{}^{\left(\alpha \right)}(r) \end{array}\right.$$

where $\Psi_{b,n}$ and $\Psi_{l,i}$ are the structural wave functions, which are also the exact solution of Equation (1). $\in_{m}$ is the acoustic wave function contribution coefficient, and $\xi_{n}$ and $\zeta_{l}$ are the structural wave function contribution coefficients. ${\hat{p}}_{q}$ is the particular solution of the nonhomogeneous Helmholtz equation.

(8) $$\Psi_{b,n}=e^{k_{n}\tau },k_{n}=\left\{-jk_{b},jk_{b},-k_{b},k_{b}\right\}$$

(9) $$\Psi_{l,i}=\left\{e^{jk_{l}\tau },e^{-jk_{l}\tau }\right\}$$

Since there is a direct coupling between the acoustic cavity and structure, ${\tilde{w}}_{a}$ represent the particular solution induced by the sound pressure inside the cavity. The additional terms, ${\tilde{w}}_{F}$ and ${\tilde{w}}_{q}$, are the particular solution caused by the existence of external excitation and acoustic source, respectively.

For the acoustic cavity, $\Phi_{m}$ is the acoustic wave function, which satisfies the Helmholtz equation and is defined as:

(10) $$\Phi_{m}(x,y)=\left\{ \begin{array}{l} \Phi_{m,a}=\cos(k_{x,a}\cdot x)\cdot e^{-jk_{y,a}y}\\ \Phi_{m,b}=e^{-jk_{x,b}x}\cdot \cos(k_{y,b}\cdot y) \end{array}\right.$$

(11) $$\left(k_{x,a},k_{y,a}\right)=\left\{ \begin{array}{l} \left(\frac{a\pi }{L_{x}},+\sqrt{k^{2}-{\left(\frac{a\pi }{L_{x}}\right)}^{2}}\right)\\ \left(\frac{a\pi }{L_{x}},-\sqrt{k^{2}-{\left(\frac{a\pi }{L_{x}}\right)}^{2}}\right) \end{array}\right.a=0,1,2,\cdots$$

(12) $$\left(k_{x,b},k_{y,b}\right)=\left\{ \begin{array}{l} \left(+\sqrt{k^{2}-{\left(\frac{b\pi }{L_{y}}\right)}^{2}},\frac{b\pi }{L_{y}}\right)\\ \left(-\sqrt{k^{2}-{\left(\frac{b\pi }{L_{y}}\right)}^{2}},\frac{b\pi }{L_{y}}\right) \end{array}\right.b=0,1,2,\cdots$$

**Figure 6:**
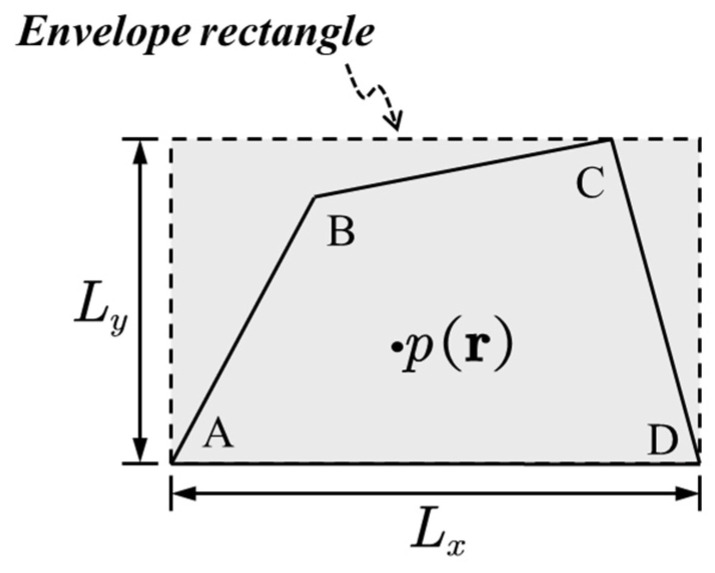
Enveloping rectangle of an irregular shaped acoustic cavity.

Due to the irregularity of the acoustic cavity, the wave functions are defined in the envelope rectangle shown in Figure 6.

Corresponding to each acoustic wave function, the cavity sound pressure induced transverse displacement of the plate can be expressed as

(13) $$\left\{ \begin{array}{r} {\tilde{w}}_{a,m}(\tau^{\prime })=e^{\left(-jk_{y,m}(y_{0}+\tau^{\prime }\cdot \sin\alpha )\right)}(\frac{\left(C_{1}-k_{b}^{4})\cdot \cos(k_{x,m}\cdot (x_{0}+\tau^{\prime }\cos\alpha )\right)}{D\left[{\left(C_{1}-k_{b}^{4}\right)}^{2}+C_{2}^{2}\right]}\\ -\frac{C_{2}\cdot \sin(k_{x,m}\cdot (x_{0}+\tau^{\prime }\cos\alpha ))}{D\left[{\left(C_{1}-k_{b}^{4}\right)}^{2}+C_{2}^{2}\right]})\\ {\tilde{w}}_{a,m}(\tau^{\prime })=e^{\left(-jk_{x,m}(y_{0}+\tau^{\prime }\cdot \cos\alpha )\right)}(\frac{\left(C_{1}-k_{b}^{4})\cdot \cos(k_{y,m}\cdot (y_{0}+\tau^{\prime }\sin\alpha )\right)}{D\left[{\left(C_{1}-k_{b}^{4}\right)}^{2}+C_{2}^{2}\right]}\\ -\frac{C_{2}\cdot \sin(k_{y,m}\cdot (y_{0}+\tau^{\prime }\sin\alpha ))}{D\left[{\left(C_{1}-k_{b}^{4}\right)}^{2}+C_{2}^{2}\right]}) \end{array}\right.$$

where

(14) $$\left\{ \begin{array}{l} C_{1}=\frac{1}{2}[{\left(k_{x,m}\cdot \cos\alpha +k_{y,m}\cdot \sin\alpha \right)}^{4}+{\left(-k_{x,m}\cdot \cos\alpha +k_{y,m}\cdot \sin\alpha \right)}^{4}]\\ C_{2}=\frac{j}{2}[{\left(-k_{x,m}\cdot \cos\alpha +k_{y,m}\cdot \sin\alpha \right)}^{4}-{\left(k_{x,m}\cdot \cos\alpha +k_{y,m}\cdot \sin\alpha \right)}^{4}] \end{array}\right.$$

When the panel is subjected to a concentrated point force $F_{0}\left(\tau =\tau_{F}\right)$, the particular solution is [31]

(15) $${\tilde{w}}_{F}(\tau )=\frac{-jF_{0}}{4Dk_{b}^{4}}\left(e^{-jk_{b}\left|\tau -\tau_{F}\right|}-j\cdot e^{-k_{b}\left|\tau -\tau_{F}\right|}\right)$$

**Figure 7:**
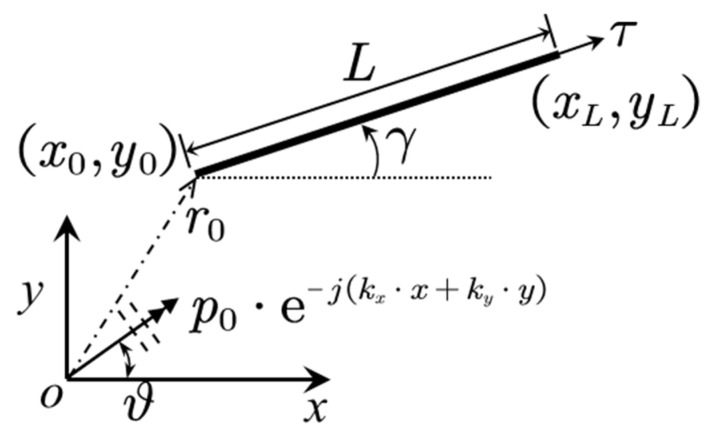
A straight plate under plane wave excitation.

In this paper, because the sandwich panel is subjected to the acoustic plane wave excitation, as shown in Figure 7, the particular solution for a single flat panel can be expressed as follows [31]:

(16) $$\begin{array}{ll} {\tilde{w}}_{F}(\tau^{\prime })= & \frac{-jp_{0}}{4Dk_{b}^{3}}[\frac{i(e^{-i(\tau^{\prime }k_{b}+x_{0}k_{x}+y_{0}k_{y})}-e^{-i(k_{x}(\tau^{\prime }\cos(\gamma )+x_{0})+k_{y}(\tau^{\prime }\sin(\gamma )+y_{0})})}{k_{b}-\cos(\gamma )k_{x}-\sin(\gamma )k_{y}}\\ & -\frac{e^{-i(x_{0}k_{x}+y_{0}k_{y})}(e^{-\tau^{\prime }k_{b}}-e^{-i\tau^{\prime }\cos(\gamma )k_{x}+\sin(\gamma )k_{y})})}{ik_{b}+\cos(\gamma )k_{x}+\sin(\gamma )k_{y}}\\ & +\frac{i(e^{-i(k_{b}(L-\tau^{\prime })+k_{x}x_{L}+k_{y}y_{L})}-e^{-i(k_{x}(\tau^{\prime }\cos(\gamma )+x_{0})+k_{y}(\tau^{\prime }\sin(\gamma )+y_{0})})}{k_{b}+\cos(\gamma )k_{x}+\sin(\gamma )k_{y}}\\ & -\frac{i(e^{-i(k_{x}(\tau^{\prime }\cos(\gamma )+x_{0})+k_{y}(\tau^{\prime }\sin(\gamma )+y_{0}))}-e^{k_{b}(\tau^{\prime }-L)-i(k_{x}x_{L}+k_{y}y_{L})})}{k_{b}+i(\cos(\gamma )k_{x}+\sin(\gamma )k_{y})}] \end{array}$$

**Figure 8:**
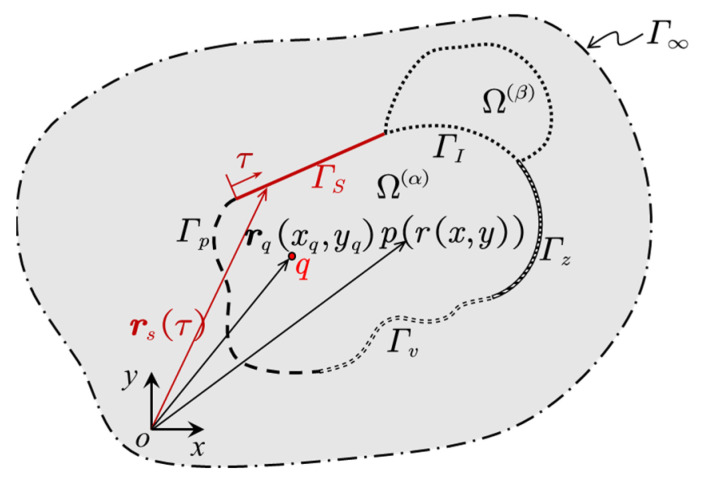
A complete vibro-acoustic system.

Since there are direct couplings between the sandwich structure and the interior acoustic cavities, Figure 8 gives a simple demonstration of a panel–cavity coupled system which consists of two adjacent sub-domains $\Omega^{\left(\alpha \right)}$ and $\Omega^{\left(\beta \right)}$.

Take sub-domain $\Omega^{\left(\alpha \right)}$ as an example, to enforce the boundary condition, a weighted residual formulation is used.

(17) $$\begin{array}{l} \int_{\Gamma_{v}}W^{\left(\alpha \right)}(r)\cdot R_{v}^{\left(\alpha \right)}(r)ds+\int_{\Gamma_{p}}-℘(W^{\left(\alpha \right)}(r))\cdot R_{p}^{\left(\alpha \right)}(r)ds\\ +\int_{\Gamma_{Z}}W^{\left(\alpha \right)}(r)\cdot R_{z}^{\left(\alpha \right)}(r)ds\\ +\int_{\Gamma_{s}}W^{\left(\alpha \right)}(r)\cdot R_{s}^{\left(\alpha \right)}(r)ds\\ +\sum_{\beta =1,\beta \neq \alpha }^{N_{\Omega }}\int_{\Gamma_{I}}W^{\left(\alpha \right)}(r)\cdot R_{I}^{\left(\alpha ,\beta \right)}(r)ds=0 \end{array}$$

In this sub-domain, there are five kinds of boundary condition: $\Gamma_{v}$ is the particle velocity boundary condition (including acoustic rigid wall), $\Gamma_{p}$ is the sound pressure boundary condition, $\Gamma_{Z}$ is the acoustic impedance boundary condition, $\Gamma_{s}$ is the vibro-acoustic coupling boundary condition, and $\Gamma_{I}$ is the acoustic continuity boundary condition. On each kind of boundary, the residual function is defined as

(18) $$\left\{ \begin{array}{l} \gamma \in \Gamma_{v}:R_{v}^{\left(\alpha \right)}(r)=℘({\hat{p}}^{\left(\alpha \right)}(r))-v_{n}^{*}(r)\\ \gamma \in \Gamma_{p}:R_{p}^{\left(\alpha \right)}(r)=\hat{p}(r)-p^{*}(r)\\ \gamma \in \Gamma_{z}:R_{z}^{\left(\alpha \right)}(r)=℘(\hat{p}(r))-\frac{\hat{p}(r)}{Z_{n}^{*}(r)}\\ \gamma \in \Gamma_{s}:R_{s}^{\left(\alpha \right)}(r)=℘(\hat{p}(r))-n_{s}\cdot n^{\left(\alpha \right)}\cdot j\omega {\hat{w}}_{s}(r)\\ \gamma \in \Gamma_{I}:R_{I}^{\left(\alpha ,\beta \right)}(r)=\left(℘({\hat{p}}^{\left(\alpha \right)}(r))-\frac{{\hat{p}}^{\left(\alpha \right)}(r)}{Z_{\mathrm{int}}}\right)+\left(℘({\hat{p}}^{\left(\beta \right)}(r))+\frac{{\hat{p}}^{\left(\beta \right)}(r)}{Z_{\mathrm{int}}}\right) \end{array}\right.$$

The linear differential operator ℘(·) is defined as

(19) $$℘(\cdot )=\frac{j}{\rho_{0}\omega }\frac{\partial \cdot }{\partial n}$$

where $\rho_{0}$ is the density of the acoustic medium. According to the conventional Galerkin procedure, the weighted function can also be written as a summation of the same acoustic wave function given in Equation (10):

(20) $$W(r)=\sum_{m=1}^{m_{a}}{\tilde{\in }}_{m}\cdot \Phi_{m}(r)$$

Substituting Equations (7) and (19) into the boundary weighted residual formulation (17) leads to the linear equations of the acoustic cavity:

(21) $$\left[M_{AA}M_{AS}]\cdot [\eta ]=[b\right]$$

(22) $$M_{AA}\;=\;\left[\begin{array}{cccc} A^{\left(1,1\right)} & C_{p}^{\left(1,2\right)} & \cdots & C_{p}^{\left(1,N_{a}\right)}\\ C_{p}^{\left(2,1\right)} & A^{\left(2,2\right)} & \cdots & C_{p}^{\left(2,N_{a}\right)}\\ \vdots & \vdots & \ddots & \vdots \\ C_{p}^{\left(N_{a},1\right)} & C_{p}^{\left(N_{a},2\right)} & \cdots & A^{\left(N_{a},N_{a}\right)} \end{array}\right]$$

(23) $$M_{AS}\;=\;\left[\begin{array}{cccc} C_{sp}^{\left(1,1\right)} & C_{sp}^{\left(1,2\right)} & \cdots & C_{sp}^{\left(1,N_{s}\right)}\\ C_{sp}^{\left(2,1\right)} & A_{sp}^{\left(2,2\right)} & \cdots & C_{sp}^{\left(2,N_{s}\right)}\\ \vdots & \vdots & \ddots & \vdots \\ C_{sp}^{\left(N_{a},1\right)} & C_{sp}^{\left(N_{a},2\right)} & \cdots & A_{sp}^{{}^{\left(N_{a},N_{s}\right)}} \end{array}\right]$$

where

(24) $$\left[\eta \right]={\left[\in^{\left(1\right)}{}_{}\in^{\left(2\right)}{}_{}\cdots_{}\in^{\left(N_{a}\right)}{}_{}\xi^{\left(1\right)}{}_{}\xi^{\left(2\right)}{}_{}\cdots_{}\xi^{\left(N_{s}\right)}\right]}^{T}$$

(25) $$\left[b\right]={\left[\begin{array}{cccc} b^{\left(1\right)} & b^{\left(2\right)} & \cdots & b^{\left(N_{a}\right)} \end{array}\right]}^{T},b^{{}^{\left(\alpha \right)}}=f^{\left(\alpha ,\alpha \right)}+\sum_{\beta =1,\beta \neq \alpha }^{N_{a}}f^{\left(\alpha ,\beta \right)}$$

where $N_{a}$ and $N_{s}$ are the total number of acoustic cavities and sub-plates, A is the boundary condition matrix, $C_{sp}$ is the vibro-acoustic coupling matrix, and $C_{p}$ is the coupling matrix between adjacent acoustic cavities; the details of those matrices are given in Appendix A. Assuming the total number of acoustic wave functions is $m_{a}$, f is a $m_{a}\times 1$ vector related to the predefined boundary conditions, and the detailed formulation can also be found in the Appendix. To balance the computational efficiency and accuracy, the number of acoustic wave functions can be determined by using the structural bending wave number:

(26) $$\frac{n_{a}}{L_{x}}\approx \frac{n_{b}}{L_{y}}\geq \frac{4}{\lambda_{b}}=\frac{2\cdot k_{b}}{\pi }$$

Associating with the structural boundary condition, the dynamic equations of the structural part of the system can also be written in a simple matrix form:

(27) $$\left[\begin{array}{ccc} M_{SA} & M_{SB} & M_{SL} \end{array}]\cdot [\eta^{\prime }]=[b_{S}\right]$$

where

(28) $$\left[\eta^{\prime }\right]={\left[\begin{array}{cccccccccccc} \in^{\left(1\right)} & \in^{\left(2\right)} & \cdots & \in^{\left(N_{a}\right)} & \xi^{\left(1\right)} & \xi^{\left(2\right)} & \cdots & \xi^{\left(N_{s}\right)} & \zeta^{\left(1\right)} & \zeta^{\left(2\right)} & \cdots & \zeta^{\left(N_{s}\right)} \end{array}\right]}^{T}$$

The sub-matrices $M_{SA}$, $M_{SB}$ and $M_{SL}$ are the vibro-acoustical coupling matrix, structural bending coefficient matrix, and structural in-plane coefficient matrix, respectively. Thus, the complete form of the governing equation of the system can be written as

(29) $$\left[\begin{array}{ccc} M_{AA} & M_{SB} & 0\\ M_{SA} & M_{SB} & M_{SL} \end{array}\right]\cdot \left[\begin{array}{c} \in^{\left(1\right)}\\ \vdots \\ \in^{\left(N_{a}\right)}\\ \xi^{\left(1\right)}\\ \vdots \\ \xi^{\left(N_{s}\right)}\\ \zeta^{\left(1\right)}\\ \vdots \\ \zeta^{\left(N_{s}\right)} \end{array}\right]=\left[\begin{array}{l} b\\ b_{S} \end{array}\right]$$

As the key indicator of evaluating the sound insulation performance of the sandwich panel, the sound transmission loss (STL) is used to be the objective of the optimization procedure:

(30) $$\mathrm{STL}=10\cdot \log_{{}_{10}}\left(\frac{W_{inc}}{W_{rad}}\right)$$

where $W_{inc}$ and $W_{rad}$ are the incident and radiation sound power, respectively.

**Figure 9:**
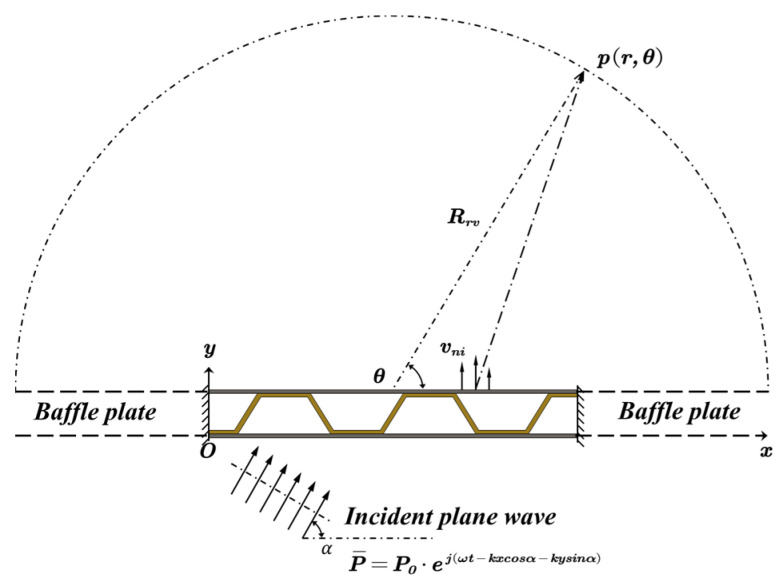
Sound transmission through the sandwich panel.

As shown in Figure 9, the external excitation is incident plane sound wave with an angle of α. The radiation sound pressure can be calculated by using the Raleigh’s integral [32]:

(31) $$p\left(x,\theta \right)=\int_{0}^{L}\frac{\rho_{0}\omega }{2}\cdot H_{0}^{\left(2\right)}\left(k\cdot \sqrt{\left(R_{re}\cos\theta -x\right)+(R_{re}\sin\theta )}\right)\cdot v_{ni}\left(x\right)dx$$

where: $\rho_{0}$ is the density of the acoustic medium, $H_{0}^{\left(2\right)}$ is the Hankel function of the second kind, $R_{re}$ is the radius of the half-circle observation surface, $v_{ni}$ is the normal velocity, and k is the wave number of the acoustic wave.

Thus, the radiation sound power can be obtained as follows:

(32) $$W_{rad}=\frac{1}{2\rho_{0}c_{0}}\int_{half\;circle}p^{2}(x,\theta )\cdot R_{re}d\theta$$

Since the sound transmission loss of the sandwich panel varies with the target frequency, in order to evaluate the sound insulation performance in a specific frequency range $\left(\omega_{1}\sim \omega_{2}\right)$, the frequency averaged STL used in the optimization process:

(33) $$\mathrm{STL}=\frac{1}{\omega_{2}-\omega_{1}}\cdot \int_{\omega_{1}}^{\omega_{2}}\mathrm{STL}\underset{}{}d\omega$$

### 2.3. Numerical Verification

**Figure 10:**
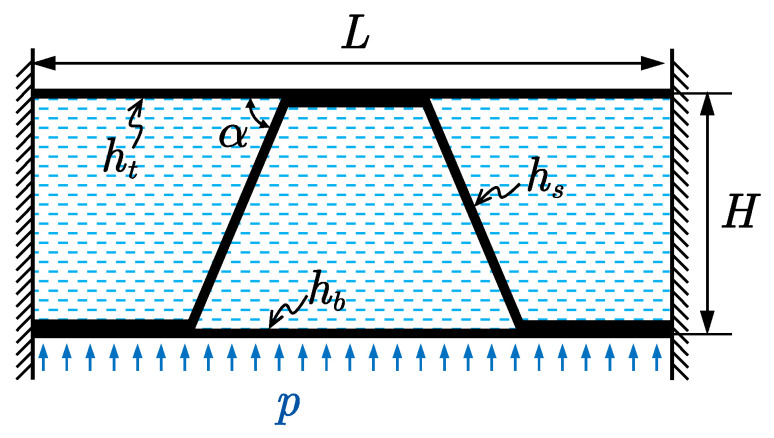
A vibro-acoustic model of a unit cell of the sandwich plate with corrugated core.

Consider a unit cell of the sandwich plate with corrugated core, as shown in Figure 10, the total length of the structure is *L* = 0.5 m, the distance between the top face sheet and bottom face sheet is *H* = 0.15 m, the inclined angle of the core plate α = 60°. The two face plates and the core sheet have the same thickness, which is *h_t_* = *h_b_* = *h_s_* = 2 mm.

This vibro-acoustic coupling system contains three trapezoidal acoustic cavities, which are filled with air. Each acoustic cavity is coupled with the plates occupying its boundaries. Both structural ends of the unit cell are clamped, and the left and right end boundaries of the acoustic cavity are considered as acoustically rigid wall. Assuming there is a uniformly distributed pressure *p* with an unit magnitude acting on the bottom sheet, to verify the reliability and computational efficiency of the wave-based method, both the conventional finite element method (ANSYS^®^) and the WBM are used to calculate the vibro-acoustic response of the system.

**Figure 11:**
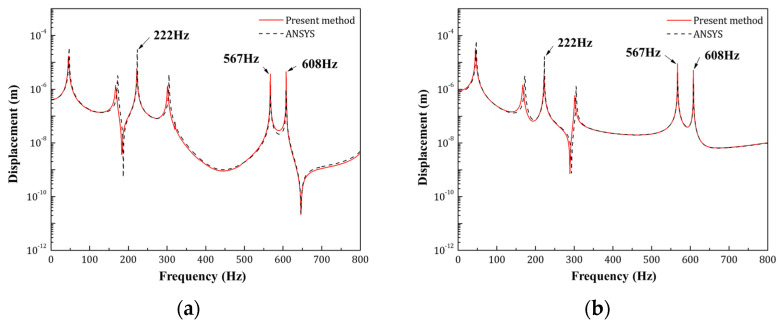
The displacement response of the observation points on the upper face sheet: (**a**) *L*/3 of the top face sheet; (**b**) *L*/2 of the top face sheet.

Figure 11 presents the displacement response of two observation points on the top face sheet in the frequency range of 1–800 Hz.

**Figure 12:**
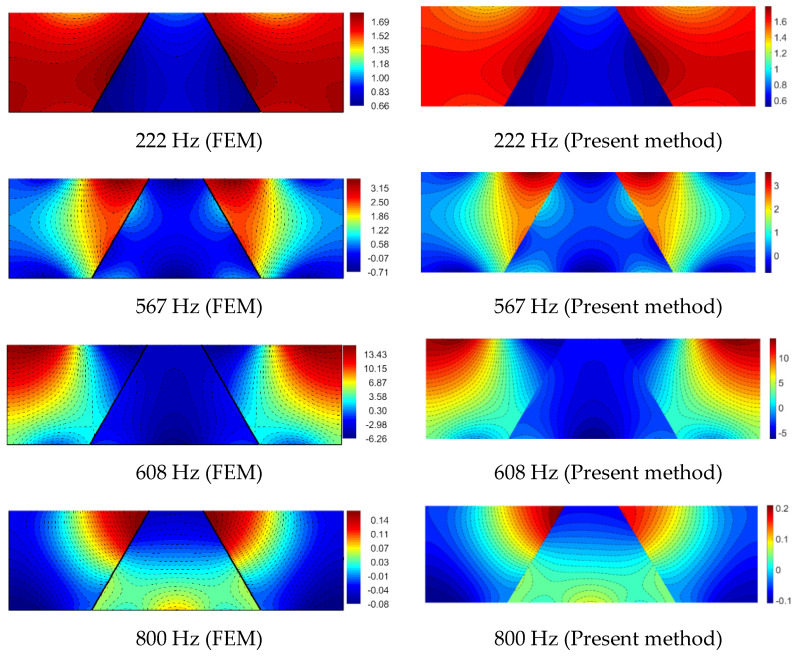
The sound pressure distribution response of the acoustic cavities.

Accordingly, Figure 12 gives the sound pressure distribution of the acoustic cavities at the resonance frequencies marked in Figure 11.

Both the structural displacement response and the in-cavity sound pressure distribution indicate that the result obtained by the WBM agrees very well with the conventional finite element method. Especially for the in-cavity sound pressure response, not only the pressure distribution but also the magnitude has a great match with the FEM commercial software.

The comparison between the conventional FEM and the present method proves the reliability of the WBM. According to the truncation rules of the WBM, the total number of basic wave functions used to approximate the field variables are related to the plate bending wavelength, which means the number of wave functions would become larger when the targeting frequency increasing.

**Figure 13:**
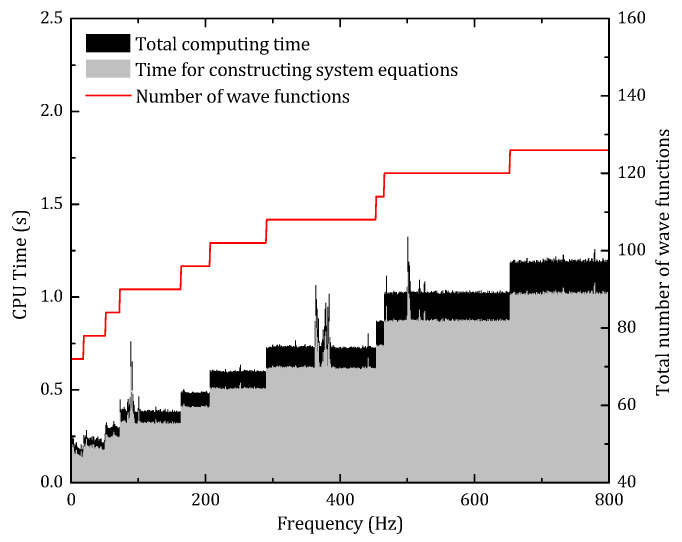
The CPU time and system DOFs of present method with respect to frequency.

**Figure 14:**
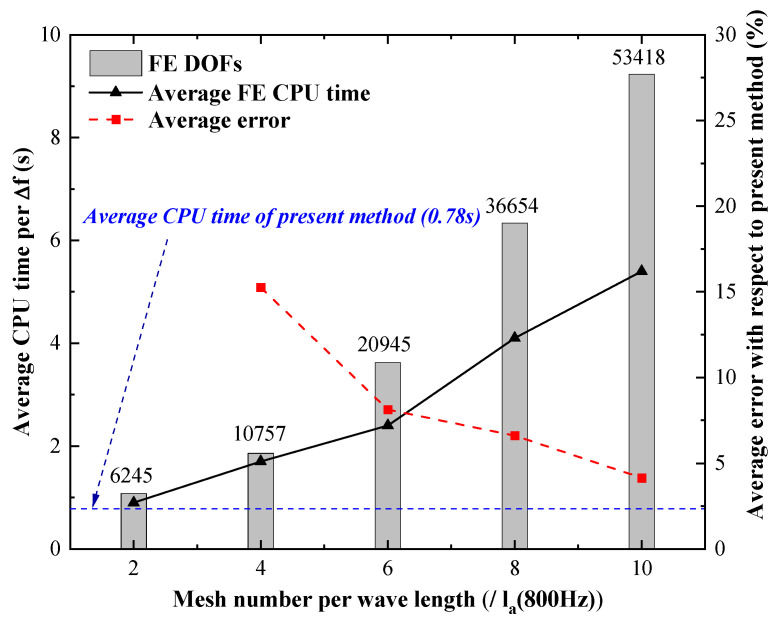
The calculation time and relative error (with respect to the present method) of conventional FEM.

As shown in Figure 13, both the number of wave functions and computational central processing unit (CPU) time are proportional to the frequency. Moreover, since the WBM is frequency-dependent, the system equation should be reconstructed in each frequency step, which costs most of the computational time. Nonetheless, at the highest frequency, 800 Hz, the total DOFs of the system is 127, which is still much smaller than the conventional FEM. Figure 14 presents the frequency average CPU time of the present method, and FEM, the relative error between the two methods, is represented by red square dots.

For FEM, the comparison result indicates that with the increase of the mesh number per wavelength, the system DOFs is strikingly enlarged, and the computational accuracy is significantly improved. From the perspective of relative error, because of the analytical nature of the WBM, the result of the FEM would converge to the result of the WMB. When the relative error reduces to less than 5%, the average CPU time of FEM is 5.4 s, which is almost seven times that of the present method.

According to the validation results presented above, the WBM is reliable in solving the dynamic response of the vibro-acoustic coupling system and has much higher computational efficiency than the conventional FEM. Those advantages make the WBM a good choice for the vibro-acoustic optimization procedure, which usually requires enormous computation.

## 3. Structural–Acoustic Optimization

**Figure 15:**
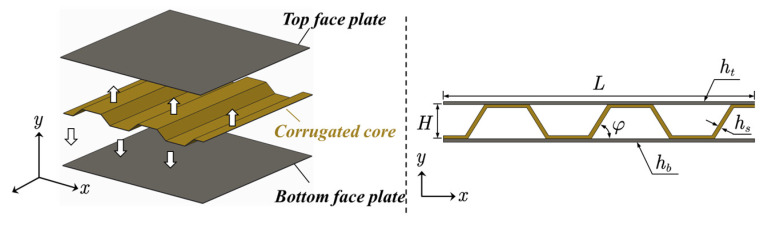
The sandwich panel model used in the optimization.

The geometry configuration of the target model is shown in Figure 15; the sandwich panel has a total length of *L* = 0.7 m, and the thickness of the core layer (the distance between the two face sheets) is *H* = 0.04 m. The corrugated core has five inclined stiffeners (lateral side of the trapezoid) and the base angle of the hypotenuse is *φ*. The thickness of the top panel, core sheet, and bottom panel are *h_t_*, *h_s_*, and *h_b_*, respectively. The external excitation is incident plane sound wave with an angle of α = 45°. In the x-direction, both ends of the sandwich panel are clamped and infinitely baffled.

### 3.1. The Baseline Model

Before performing the optimization, a baseline model should be proposed to appraise the optimization result. To ensure the rationality of the baseline model, a parametric study with respect of the inclined angle *φ* is applied. In the parametric study, the top face panel, the core sheet, and the bottom face panel are assumed to have the same material and thickness, thus, *h_t_* = *h_b_* = *h_s_* = 2 mm. The whole sandwich panel is made of aluminum, and the material properties are listed in Table 1.

**Figure 16:**
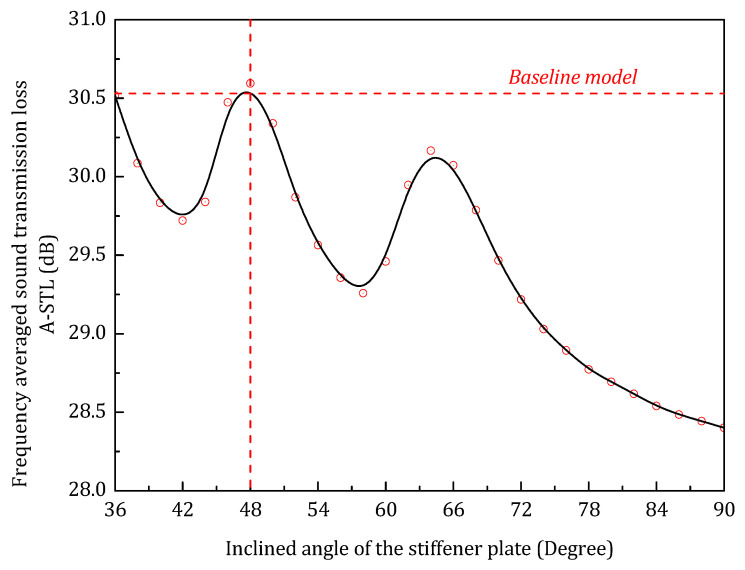
Frequency averaged sound transmission loss with respect to the inclined angle of the stiffener panel.

In the frequency range 25–1200 Hz, the frequency averaged sound transmission loss variation with respect to the core inclined angle is illustrated in Figure 16.

As shown in the figure, when the core stiffener has an inclined angle of 48 degrees, the sandwich panel has the largest STL_avg_ of 30.5 dB, which can be treated as the single-parameter optimal geometrical configuration. Table 2 gives the details of the baseline model. *m* is the mass of baseline model.

**Figure 17:**
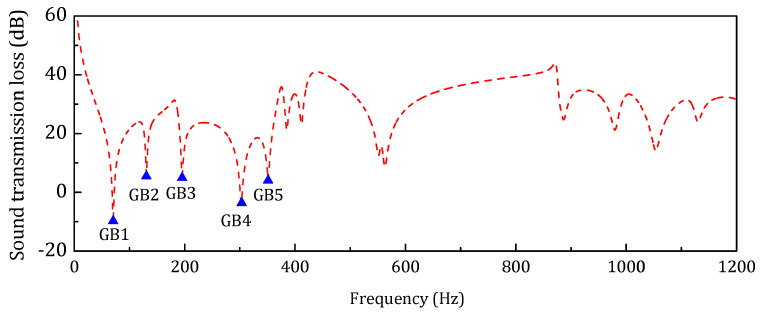
STL spectrum of the baseline model (GB, Global bending mode).

In Table 2, *f*_1_ and *m* are the first resonance frequency and total mass of the sandwich structure. The spectrum of the STL in the targeting frequency range is shown in Figure 17.

According to the distribution of the resonance frequencies of the baseline model, the targeting frequency range 25–1200 Hz is separated into three frequency ranges: (1) in the low-frequency range, 25–300 Hz, only the global bending is included; (2) in the middle-frequency range, 300–800 Hz, both the global and local modes are included; and (3) in the 800–1200 Hz, almost all the local resonance modes are included.

### 3.2. Vibro-Acoustic Optimization

In order to achieve the best possible sound insulation performance, a multi-parameter optimization is implemented, and the thickness of top face plate, the core sheet, the bottom face plate, and the inclined angle of stiffener are selected as the optimization variables:

(34) $$b=\left[h_{t},h_{s},h_{b},\varphi \right]$$

The objective function is to maximize the STL_avg_ in the targeting frequency range:

(35) $$T=\max\{{\mathrm{STL}}_{\mathrm{avg}}(\langle \omega_{1},\omega_{2}\rangle ,b)\}$$

**Figure 18:**
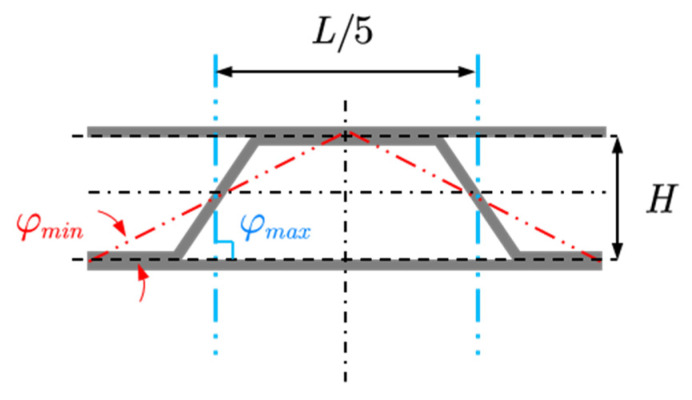
The variation ranges of the stiffener panels.

As shown in Figure 18, to ensure that the two adjacent inclined stiffeners do not interfere, the base angle of the hypotenuse *φ* is limited by the constraint as follows:

(36) $$\arctan\left(\frac{5H}{L}\right)<\varphi \leq 90^{\circ }$$

where *H* is the distance between the two face sheets, and *L* is the length of the sandwich panel.

Considering the basic topology and manufacturability of the optimization result, the ranges of value for the optimization variables are limited by the constraints as follows:

(37) $$\left\{ \begin{array}{l} 1\;\mathrm{mm}\leq h_{s}\leq 3\;\mathrm{mm}\\ 1\;\mathrm{mm}\leq h_{t}\leq 3\;\mathrm{mm}\\ h_{t}+h_{b}=4\;\mathrm{mm} \end{array}\right.$$

where *h_t_*, *h_s_*, and *h_b_* are the thickness of the top panel, core sheet, and bottom panel, respectively.

Meanwhile, to ensure not losing any lightweight property and structural stiffness, the total mass and first resonance frequency of the optimization result are subject to the constraints:

(38) $$\left\{ \begin{array}{c} f(b)-f_{0,1}\geq 0\\ m(b)-m_{0}\leq 0 \end{array}\right.$$

where $f_{0,1}$ and $m_{0}$ are the first resonance frequency and total mass of the baseline model, respectively.

The problem in this paper is a multi-parameter optimization model. For such optimization problems, due to the limitation of global searching ability, the traditional gradient-based algorithms often fall into local optimum in the optimization process and cannot converge toward the global optimum solution [17]. In order to overcome this shortcoming, the genetic algorithm with its strong global search ability is used in this paper. The genetic algorithm is a stochastic global optimization method developed by imitating the biological evolution mechanism in nature. The essence of the algorithm is to find the optimal solution to the problem through a large number of random probes. The excellent global searching ability of genetic algorithms can reduce the possibility of falling into local optimal solution, which is universal and has a wide range of applications. The genetic algorithm does not rely on the gradient information of the objective function on the parameters in the optimization process and does not require the continuous derivability of the objective function, which is suitable for large-scale and discontinuous optimization models. Although the results of the old genetic algorithm are acceptable, their main drawback is that the overall run time can easily become unacceptable. The multi-threaded genetic algorithm used in this paper can accelerate this problem because of its parallelizing [33].

The optimization is performed on Intel^®^ core^TM^ i7 CPU 11700 with 2.5 GHz 8 processor cores. MATLAB program Version R2017b is used throughout the optimization.

**Figure 19:**
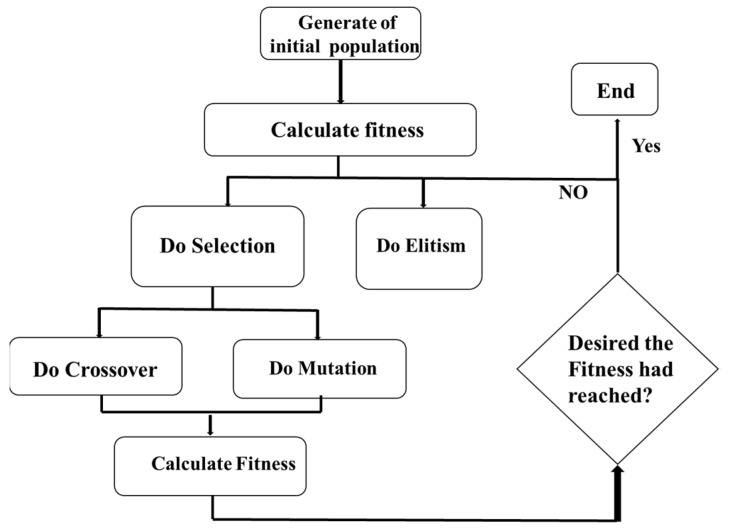
The flow chart of the genetic algorithm process.

By using the parallel computing technique, each optimization case has 10 threads, and each thread contains 100 sample individuals. To get the best solution, the values of parameters are set where the selection rate is 0.5, and the mutation rate is 0.4. To ensure the reasonability of the optimization result, at least 80 generations of evolution are performed, and considering the randomness of the genetic algorism, the final result is accepted only when the relative error of five successive generations is less than 0.1%. The program flow chart of the genetic algorithm is illustrated in Figure 19.

### 3.3. Low-Frequency Optimization

Table 3 gives the details of the optimization result in the low-frequency range.

**Figure 20:**
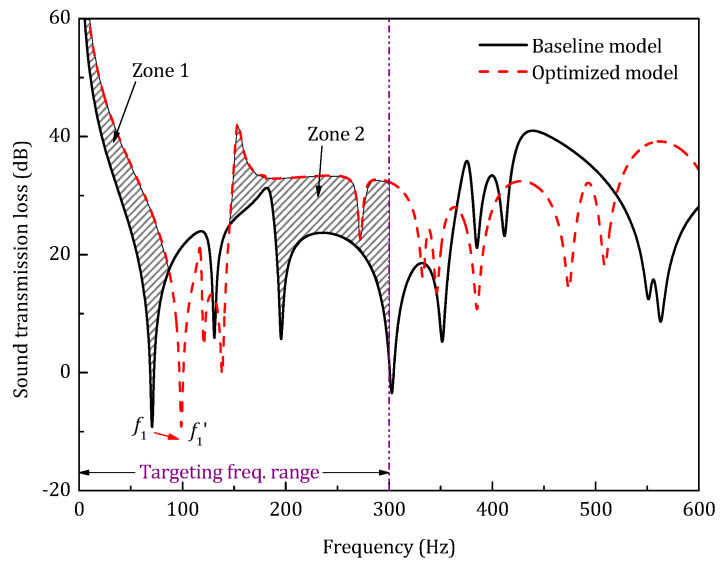
Sound transmission loss of the optimized model in low-frequency range.

According to the results listed in Table 3, the optimized model has a 7.6 dB higher STL_avg_ than the baseline model. In addition, after the optimization, the optimized model shows a 28.8 Hz increase in the first resonance frequency compared to the baseline model. Figure 20 gives the STL spectrum of the optimized model in the low-frequency range.

There are two main regions in which the STL after optimization is better than the baseline, which are marked as shadow areas. For both zone 1 and zone 2, the redistribution of the structural resonance frequencies causes the increase of the sound transmission loss. However, the total number of the resonance frequencies remains unchanged, which indicates that under current constraints (topology and total mass), the global resonance of the sandwich structure can hardly be eliminated by the optimization.

**Figure 21:**
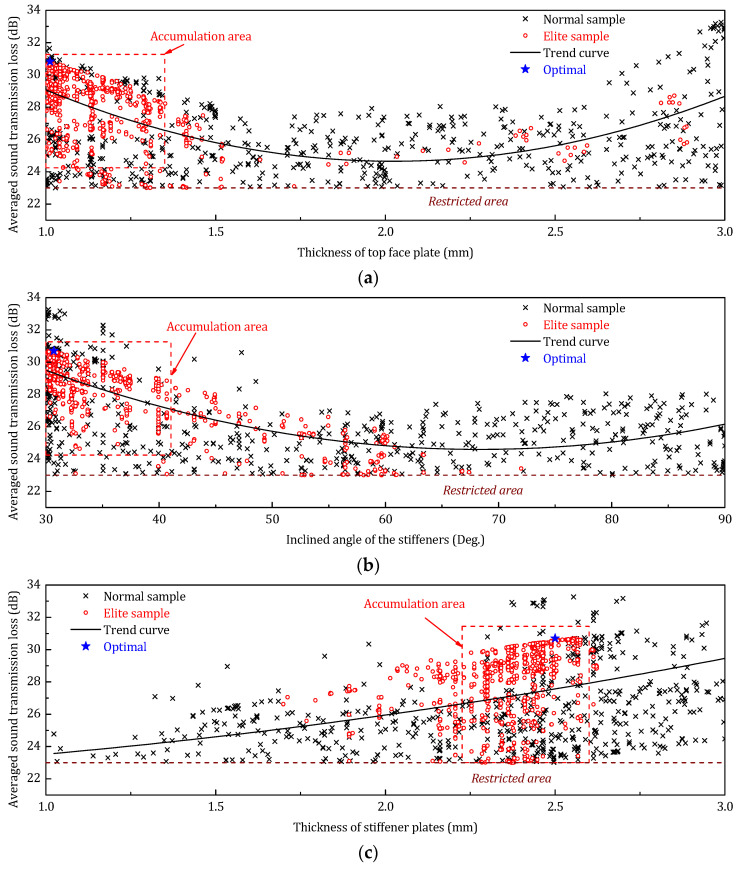
Optimization samples distribution of design parameters (low-frequency optimization). (**a**) Optimization samples distribution of *h_t_*; (**b**) Optimization samples distribution of *φ*; (**c**) Optimization samples distribution of *h_s_*.

Figure 21 shows the optimization history distribution of the STL_avg_ with respect to the three design parameters (thickness of top face plate, inclined angle of the stiffeners, and thickness of stiffener plates). The normal samples are the not retained samples in the optimization history; the elite samples are the best-retained samples; the trend curve is the trend of elite samples, and the optimal sample is the final solution of the optimization. According to the optimization sample distribution, the elite descendants of both the top panel thickness and the angle of the stiffener accumulate on the lower limit. This phenomenon indicates that in the low-frequency range, increasing the thickness of the face panel on the incident side and reducing the angle of the core stiffener can be a benefit to improving the sound insulation performance of the sandwich panel.

To determine the proportion of the variance in the sound transmission loss that is predictable from the design parameters, the coefficient of determination (COD, *R*^2^) is calculated based on the optimization history, which is defined as [34]:

(39) $$R^{2}\equiv 1-{\sum_{i}\left(y_{i}\left(x_{i}\right)-f_{i}\left(x_{i}\right)\right)}^{2}/{\sum_{i}\left(y_{i}\left(x_{i}\right)-\bar{y}\left(x\right)\right)}^{2}$$

where $y_{i}\left(x_{i}\right)$ is the elite sample point in the optimization history, $f_{i}\left(x_{i}\right)$ is the corresponding second order trend function value, $\bar{y}$ is the expectation of the sample points. The value range of the determination coefficient (*R*^2^) is 0–1. The higher the determination coefficient, the higher the influence of the change of the design parameter on the sound transmission loss.

**Figure 22:**
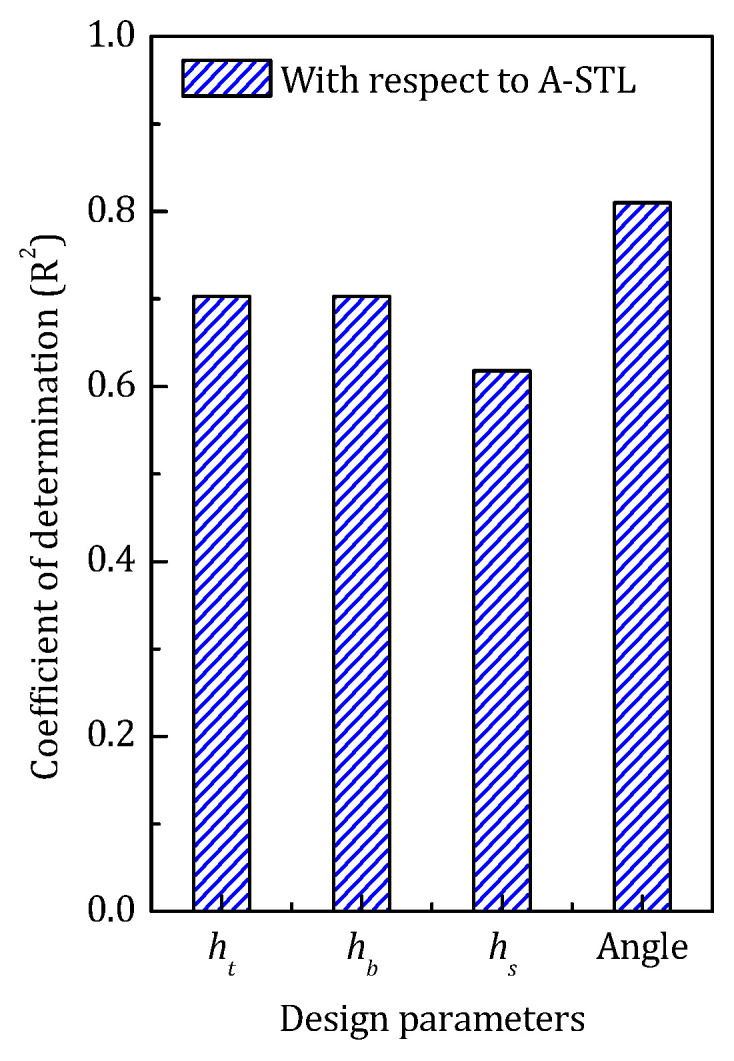
The COD of each design parameter in low-frequency range.

Figure 22 gives the coefficient of determination of each design parameter with respect to STL_avg_. The sound insulation performance of the sandwich structure mainly depends on the overall stiffness of the structure, which can be significantly influenced by geometrical configuration. All four design parameters have high COD with respect to the STL_avg_ L, and among those, the inclined angle of the stiffener has the greatest impact on the sound transmission loss of the sandwich panel.

### 3.4. Middle-Frequency Optimization

Table 4 presents the results of middle-frequency optimization. Comparing to the baseline model, the optimized model has a STL_avg_ increase of 7.9 dB, and the first resonance frequency increases by 5.1 Hz.

**Figure 23:**
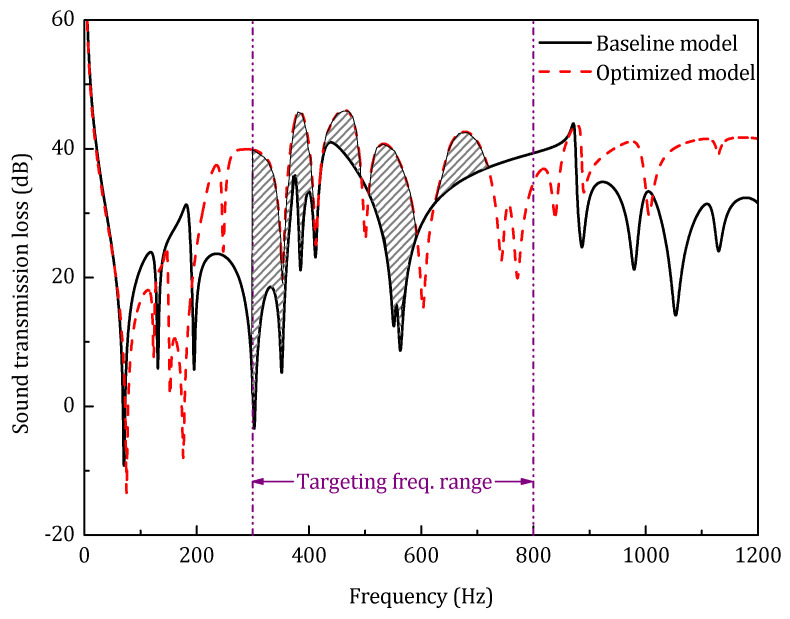
Sound transmission loss of the optimized model in mid-frequency range.

As shown in Figure 23, for the optimized model, despite there being an obvious increase of the STL_avg_ in the targeting frequency range, it has a much worse sound insulation performance in the low-frequency range. This result indicates that, under the constraints of pre-defined topology and total mass, it is hard to achieve a broadband sound insulation performance improvement by only optimizing the geometrical parameters.

**Figure 24:**
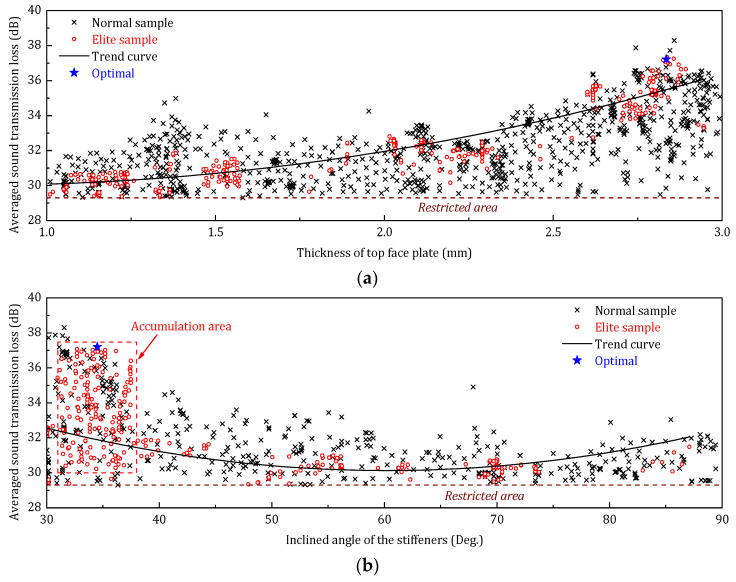

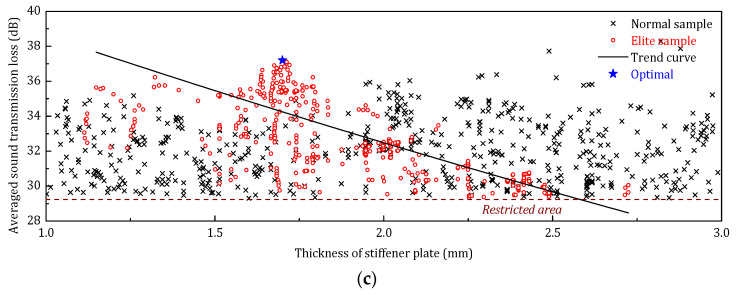
Optimization samples distribution of three design parameters (middle-frequency range). (**a**) Optimization samples distribution of *h_t_*; (**b**) Optimization samples distribution of *φ*; (**c**) Optimization samples distribution of *h_s_*.

Figure 24 shows the optimization history distribution of the STL_avg_ with respect to the three design parameters in middle-frequency range. In contrast to the low-frequency optimization, only the elite sample of the inclined angle shows a lower limit accumulation, which indicates that the less vertical stiffener is still preferred to improve the sound transmission loss of the sandwich panel.

**Figure 25:**
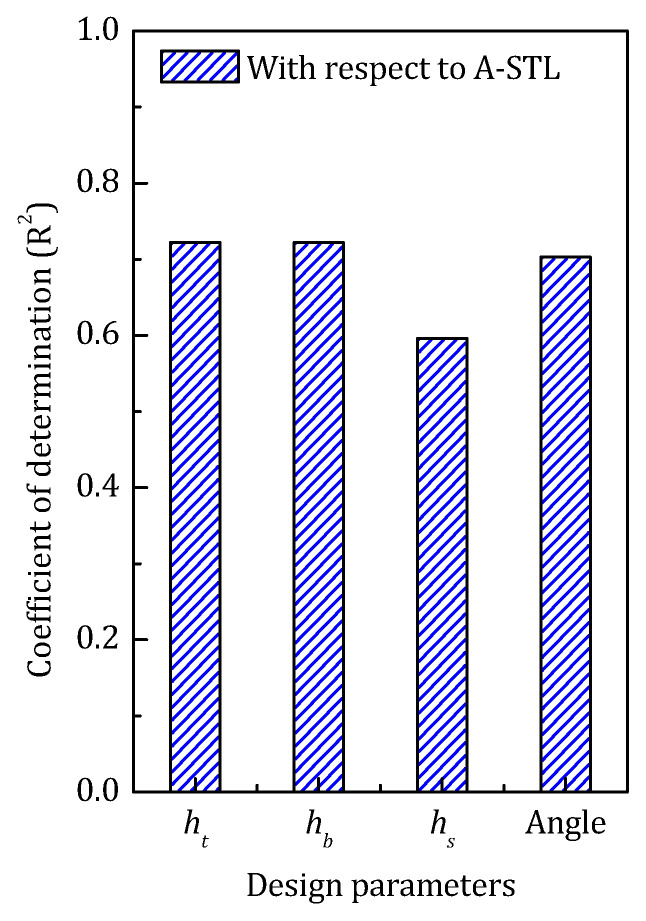
The COD of each design parameter in the middle-frequency range.

Figure 25 shows the coefficient of determination of each design parameter with respect to STL_avg_ in the middle-frequency optimization.

According to the COD result, the most important design parameters that influence the STL of the sandwich panel are still the thickness of the face sheet and the inclined angle of the stiffener. However, compared to the low-frequency optimization, with the increase of the targeting frequency range, a small rise of the COD of the face sheet thickness can be observed, and at the same time, the importance of the inclined angle of the stiffener is diminished.

### 3.5. High-Frequency Optimization

Table 5 gives the optimization results in the high-frequency range.

**Figure 26:**
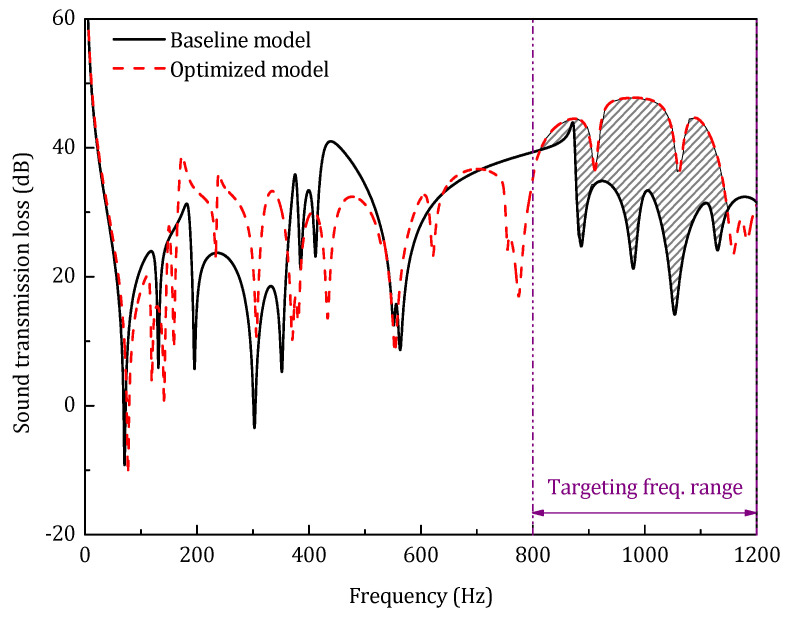
Sound transmission loss of the optimized model in the high-frequency range.

As shown in Table 5, the high-frequency optimization reduces the STL_avg_ of the corresponding frequency range by 11.7 dB, which is much more significant than the low and middle-frequency range. From the perspective of the STL spectrum illustrated in Figure 26, except for the modes redistribution, the resonance dips in the targeting frequency range are strongly diminished by the optimization.

**Figure 27:**
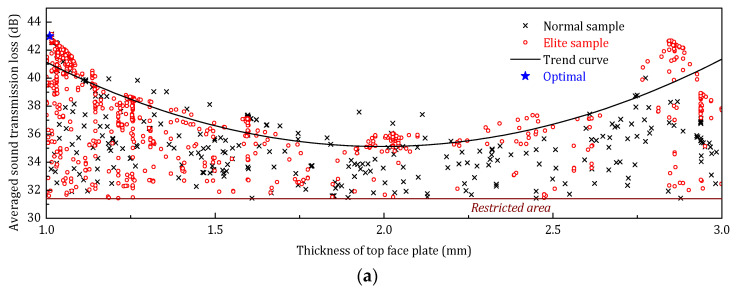

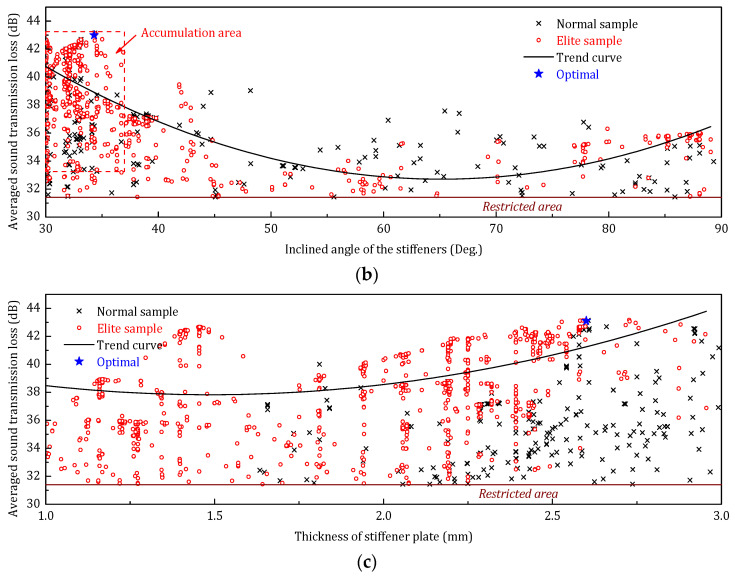
Optimization samples distribution of three design parameters (high-frequency range). (**a**) Optimization samples distribution of *h_t_*; (**b**) Optimization samples distribution of *φ*; (**c**) Optimization samples distribution of *h_s_*.

The optimization sample distribution is illustrated in Figure 27. Compared to the sample distribution of the previous two frequency ranges, the high-frequency optimization has a more evenly distribution of the elite descendants, especially for the thickness of the face panel. Nevertheless, the lower limit accumulation can also be observed in the elite sample distribution of the angle of the core sheet.

**Figure 28:**
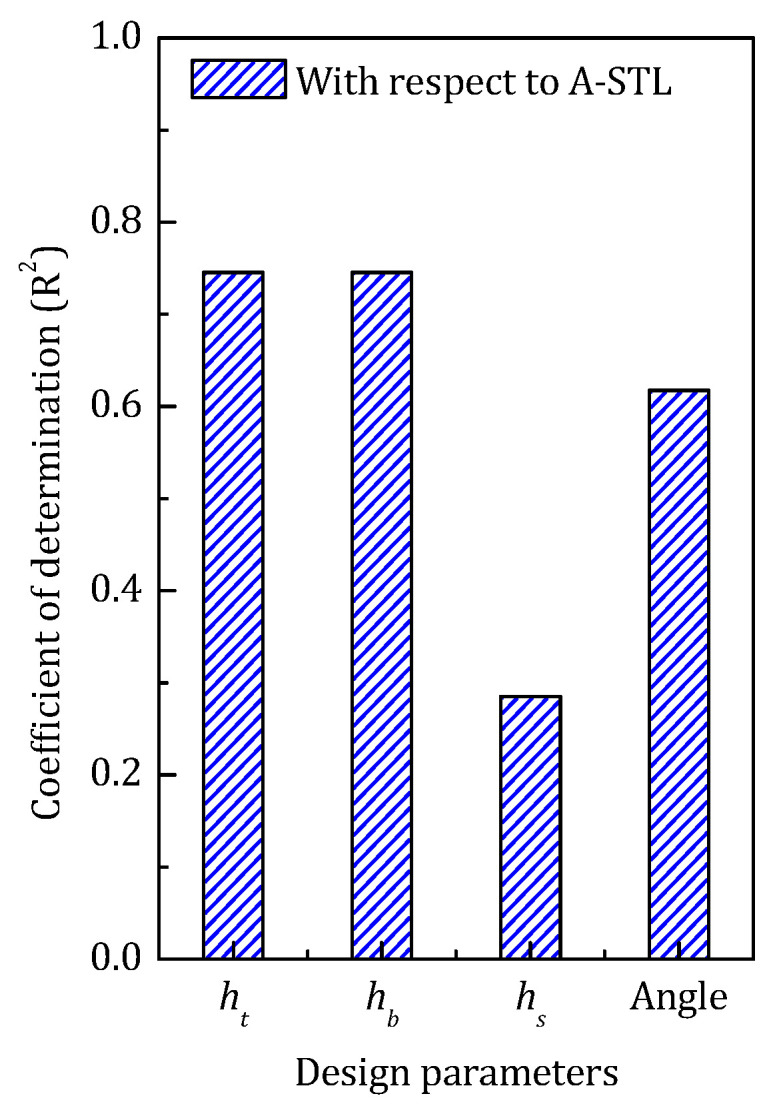
The COD of each design parameter in the high-frequency range.

The COD of the design parameters in the high-frequency optimization is shown in Figure 28. Despite the thickness of the face panel and the angle of the core stiffener still holding the dominating position, with the increase of the targeting frequency, the COD of the former one continues to rise and the latter goes the opposite way.

In the above three optimizations, the acoustic cavities in the core layer of the sandwich panel are filled with light acoustic medium (air). According to the optimization result, in addition to the thickness of the core panel (stiffener), all other three design parameters are important to the sound transmission of the sandwich panel. Meanwhile, the correlation between the design parameters and STL varies with the targeting frequency range.

**Figure 29:**
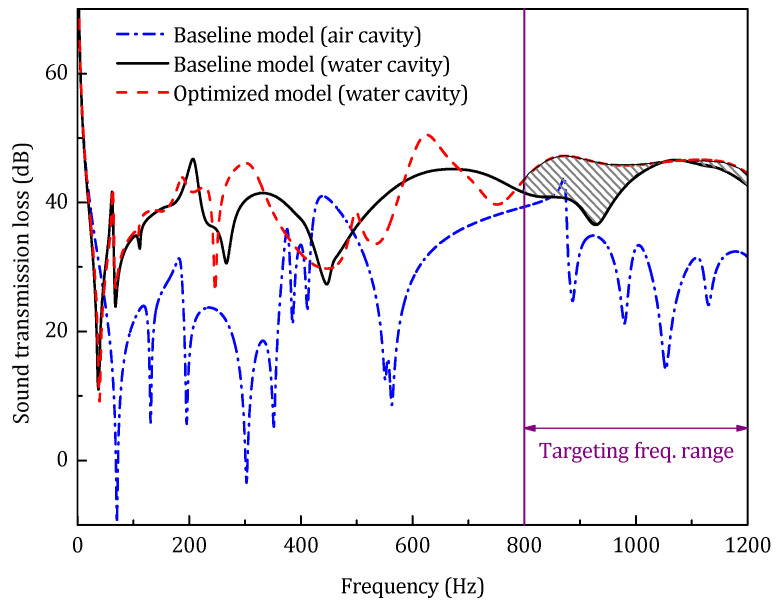
Sound transmission loss of the water cavity model.

Considering heavy acoustic medium, Figure 29 presents the optimization result of the sandwich panel with water-filled acoustic cavities in the same high-frequency range, and the results of the optimized model are listed in Table 6.

As listed in Table 6, under the same constraints, the STL_avg_ of the water cavity model only increases 3.8 dB after the structural optimization, which is far less than that in the air cavity case. This result indicates that when the equivalent stiffness of the acoustic cavity is comparable to the structural stiffness, only optimizing the structural parameters has little impact on the sound transmission characteristics of the sandwich panel. As shown in Figure 29, the filled heavy acoustic medium exhibits a strong equivalent mass effect, and the first resonance frequency of the coupled system drops from 69 Hz to 35 Hz, a drop of nearly 50%. It is precise because of this strong equivalent mass effect that the sound insulation of the water cavity model is much higher than that of the air cavity model.

**Figure 30:**
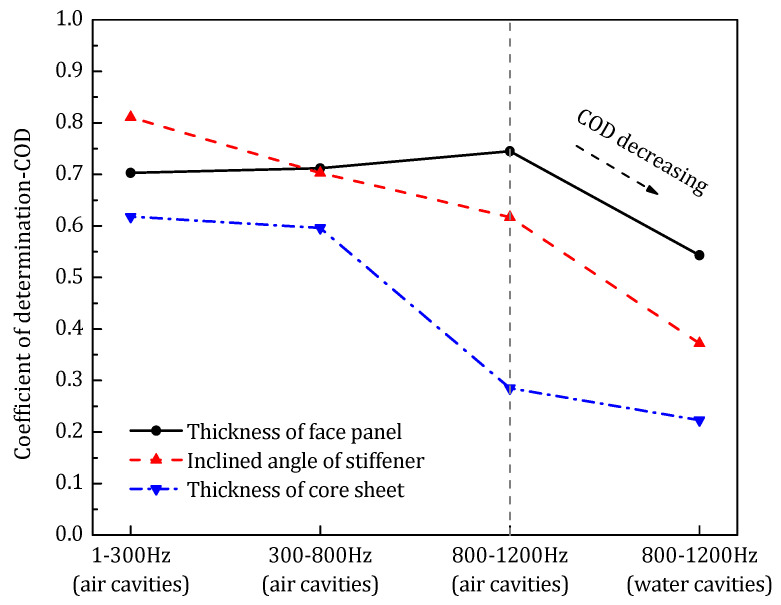
The variation of COD of design parameters with respect to STL_avg_ in different optimization models targeting different frequency ranges.

Summing up the four optimization cases, the coefficient of determination of the design parameters is given in Figure 30. As shown, firstly, with the increase of the targeting frequency, the thickness of the face panel shows an increased influence on the sound transmission loss, and instead, the parameters of the core sheet (including the thickness and the inclined angle) seem to have reduced importance on the sound insulation property of the sandwich panel. Secondly, as mentioned above, the rapid drop of the COD of the design parameters in the water cavity case also indicates that when the acoustic cavities are filled with heavy acoustic medium, the medium would dominate the sound transmission characteristics of the whole system, and changing the model configuration is not so important for the sound insulation performance.

### 3.6. Experimental Validation

**Figure 31:**
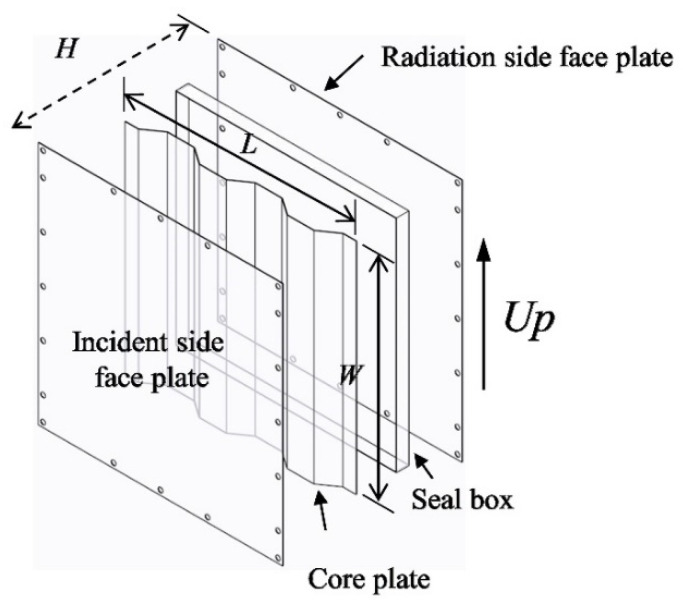
The test specimen configuration.

To validate the result in the high-frequency optimization, a sound insulation test is conducted by using the method of reverberation room and anechoic room [35]. The specimen configuration is shown in Figure 31.

Since the optimization is based on the two-dimensional cross section of the real sandwich panel, the test specimen can be treated as the stretching of the 2D cross section, and the stretching length is *W*. For comparison purposes, there are three test specimens in total, whose parameters are listed in Table 7.

Among the three test specimens, specimen 1 refers to the baseline model, and specimen 2 has different inclined angle from specimen 2. Specimen 3 is the one with the optimized cross section (due to manufacturing restrictions, the cross section of specimen 3 is not exactly the same as the calculation results), and the other two can be considered as the control group.

**Figure 32:**
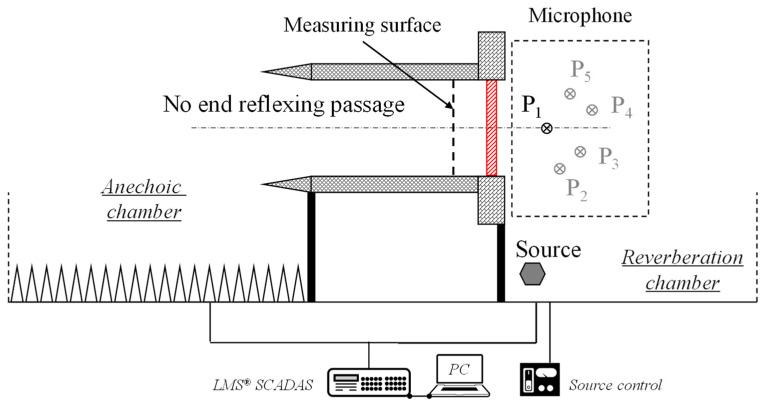
Schematics of the sound transmission loss test.

The layout of the test facility is illustrated in Figure 32; the anechoic chamber and the reverberation chamber are connected by a no end reflexing passage, and the test specimen is bolted on the test window of the reverberation chamber side. The size of the anechoic chamber is about 16 m × 11.4 m × 6.6 m, and the lower cut-off frequency is about 60 Hz. The volume of the reverberation chamber is about 268 m^3^. When the frequency is below 400 Hz, the deviation of sound pressure uniformity is less than 3 dB. When the frequency is above 400 Hz, the deviation of sound pressure uniformity is less than 1.5 dB.

**Figure 33:**
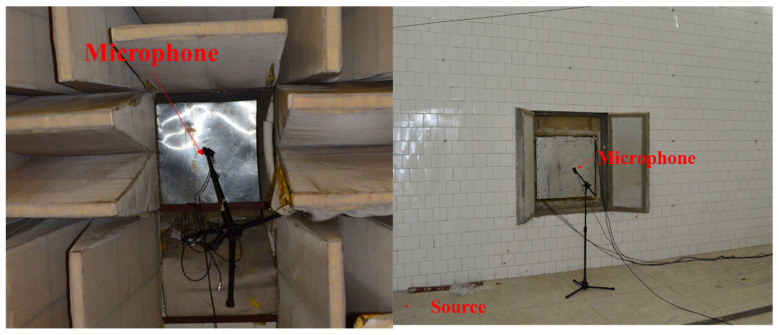
The photo of test-site in a single measuring.

In the reverberation chamber, multiple measuring positions are selected randomly to place the microphone. Subject to the experimental conditions, for the same specimen, only one microphone is used in a single test run, and after the test, the microphone is moved to another measuring position and the test is repeated under the same operating conditions. For each specimen, the test procedure is repeated 5 times, and the final sound pressure is calculated by averaging the 5 test results. On the anechoic chamber side, the same test strategy is used, and the only difference is that the 5-test position is located on the same cross section (measuring surface) of the no end reflexing passage. The photo of the test-site in given in Figure 33.

**Figure 34:**
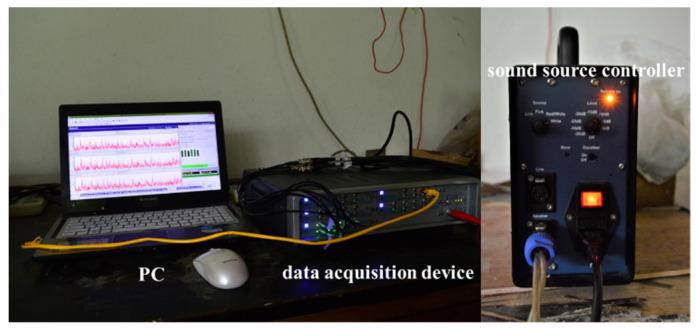
Testing equipment of sandwich plate STL experiment.

The experimental control room is located on the other side of the wall of the anechoic room and the reverberation room. All data processing and control equipment are placed in it, including sound source controller (B&K2260), power amplifier (B&K2706), data acquisition device (LMS), microphone (B&K4189), and data acquisition computer (Lenovo). In the experiment, white noise signal is used as the incident sound source, and the transmitted power is controlled by the sound source controller. Figure 34 shows the testing equipment of STL experiment.

**Figure 35:**
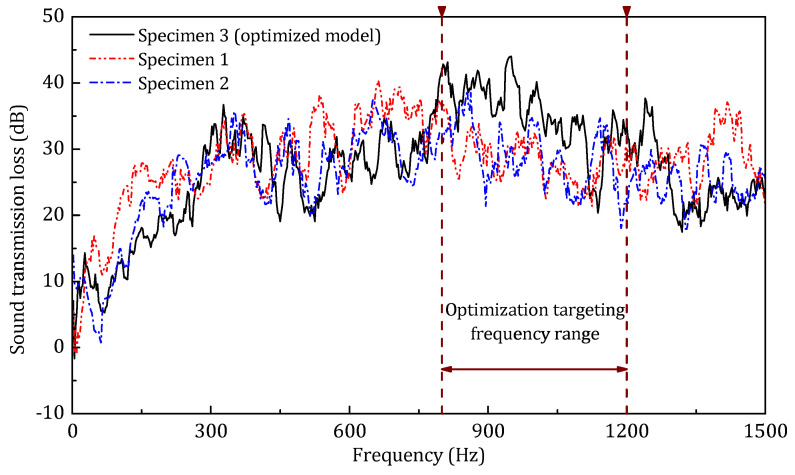
The experimental sound transmission loss of the three specimens.

The experimental results are shown in Figure 35. Comparing the STL of specimens 1 and 2, since the specimen 1 has a more inclined (core) stiffener, the sound transmission loss is correspondingly higher than that of specimen 2, especially in the low-frequency range, which agrees with the numerical prediction. According to the given curves, the optimized model (test specimen 3) has an obviously higher sound transmission loss than the other two specimens in the targeting frequency range (800–1200 Hz). This result proves the reliability of the optimization and indicates that the optimization of the 2-dimensional cross section is effective and can be applied in the practical engineering design of the sandwich panel.

## 4. Conclusions

In this paper, considering the vibro-acoustic coupling effect, a numerical model of a finite size sandwich plate with corrugated core is established by using the wave-based method. Through the numerical validation, the comparison between the WBM and conventional finite element method indicates that under the same accuracy condition, the present method can significantly reduce the system DOFs and save nearly 80% of the computational time. These advantages make the present method very suitable for calculating the objective function in an optimization problem.

(1) Together with Raleigh’s Integral, a vibro-acoustic optimization is performed by using the genetic algorithm to maximize the frequency averaged sound transmission loss (STL_avg_) of the sandwich panel in three different frequency bands.

(2) A sound insulation test is conducted by using the methods of reverberation room and anechoic room to validate the optimization results in the high-frequency range. The test data show that the optimized model has an obviously higher sound transmission loss than the control group.

Finally, the optimal results cannot be considered conclusive for all sandwich panels since they have not considered the effects of arbitrary incidence angles with respect to different frequency bandwidths, among other factors. This aspect, on which our future work will focus, cannot be neglected since, for example, the STL can be strongly dependent on the incidence angle due to coincidence effects. In addition, the method presented in this paper can be applied in the modeling of other corrugated core shapes of sandwich panels such as rectangular corrugated core or triangular corrugated core.

## Figures and Tables

**Table 1:** Model parameters of the sandwich panel.

Material Property	Value
Material density-*ρ*	2700 kg/m^3^
Young’s modulus-*E*	71 GPa
Poisson ratio-*υ*	0.33
Damping factor-*η*	0.01
Acoustic medium	Air
Acoustic medium density-*ρ*_0_	1.21 kg/m^3^
Sound speed-*c*	343 m/s

**Table 2:** Parameters of the baseline model.

Para.	*L*/m	*H*/m	*h_t_*/mm	*h_s_*/mm	*h_b_*/mm	*φ*	*f*_1_/Hz	STL_avg_/dB	*m*
	0.7	0.04	2	2	2	48°	70.4	30.56	0.438

**Table 3:** Results after optimization in low-frequency range.

	*h_t_*/mm	*h_s_*/mm	*h_b_*/mm	*φ*	*f*_1_/Hz	STL_avg_/dB	*m*
Optimized model	1	2.55	3	30.7°	99.2	30.7	0.437
Baseline model	2	2	2	48.0°	70.4	23.1	0.438

**Table 4:** Results after optimization in the middle-frequency range.

	*h_t_*/mm	*h_s_*/mm	*h_b_*/mm	*φ*	*f*_1_/Hz	STL_avg_/dB	*m*
Optimized model	2.8	1.7	1.2	34.5°	75.5	37.2	0.422
Baseline model	2	2	2	48.0°	70.4	29.3	0.438

**Table 5:** Results after optimization in the high-frequency range.

	*h_t_*/mm	*h_s_*/mm	*h_b_*/mm	*φ*	*f*_1_/Hz	STL_avg_/dB	*m*
Optimized model	1.0	2.6	3.0	34.3°	74.1	43.1	0.437
Baseline model	2	2	2	48.0°	70.4	31.4	0.438

**Table 6:** Optimization results in high-frequency range with water cavities.

	*h_t_*/mm	*h_s_*/mm	*h_b_*/mm	*φ*	*f*_1_/Hz	STL_avg_/dB	*m*
Optimized model	1.6	2.23	2.4	52.1°	37.9	46.5	0.437
Baseline model	2	2	2	48.0°	35.5	42.7	0.438

**Table 7:** Geometrical and physical parameters of the test specimens.

Specimen	*L* × *W* (m)	*H* (m)	*h_t_*/*h_b_* (mm)	*h_s_* (mm)	*φ* (Deg.)	*N_s_*	Material
1	0.70 × 0.70	0.04	2/2	2	48	5	Aluminum
2			2/2		80	5	
3			1/3	2.5	35	5	

## Data Availability

The data presented in this study are available on request from the corresponding author. The data are not publicly available due to privacy.

## Appendix A

In Equation (22):

(A1) $${\left\{A^{\left(\alpha ,\alpha \right)}\right\}}_{m_{\alpha }\times m_{\alpha }}=A_{v}^{\left(\alpha ,\alpha \right)}+A_{p}^{\left(\alpha ,\alpha \right)}+A_{Z}^{\left(\alpha ,\alpha \right)}+A_{s}^{\left(\alpha ,\alpha \right)}+A_{I}^{\left(\alpha ,\alpha \right)}$$

(A2) $$A_{v}^{\left(\alpha ,\alpha \right)}={\int_{\Gamma_{v}}\left[\Phi^{\left(\alpha \right)}\right]}^{T}\frac{j}{\rho_{0}\omega }n^{\left(\alpha \right)}\nabla \Phi^{\left(\alpha \right)}ds$$

(A3) $$A_{p}^{\left(\alpha ,\alpha \right)}=\int_{\Gamma_{p}}-\frac{j}{\rho_{0}\omega }n^{\left(\alpha \right)}\left(\nabla {\left[\Phi^{\left(\alpha \right)}\right]}^{T}\right)\Phi^{\left(\alpha \right)}ds$$

(A4) $$A_{Z}^{\left(\alpha ,\alpha \right)}={\int_{\Gamma_{z}}\left[\Phi^{\left(\alpha \right)}\right]}^{T}\frac{j}{\rho_{0}\omega }n^{\left(\alpha \right)}\nabla \Phi^{\left(\alpha \right)}-\frac{1}{Z_{n}^{*}}{\left[\Phi^{\left(\alpha \right)}\right]}^{T}\Phi^{\left(\alpha \right)}ds$$

(A5) $$A_{s}^{\left(\alpha ,\alpha \right)}=\sum_{s=1}^{N_{s}}{\int_{\Gamma_{z}}\left[\Phi^{\left(\alpha \right)}\right]}^{T}\left(\frac{j}{\rho_{0}\omega }n^{\left(\alpha \right)}\nabla \Phi^{\left(\alpha \right)}-j\omega \cdot n_{s}n^{\left(\alpha \right)}{\tilde{w}}_{a}^{\left(s\right)}\right)ds$$

(A6) $$A_{I}^{\left(\alpha ,\alpha \right)}={\int_{\Gamma_{I}}\left[\Phi^{\left(\alpha \right)}\right]}^{T}\frac{j}{\rho_{0}\omega }n^{\left(\alpha \right)}\nabla \Phi^{\left(\alpha \right)}-\frac{1}{Z_{\mathrm{int}}}{\left[\Phi^{\left(\alpha \right)}\right]}^{T}\Phi^{\left(\alpha \right)}ds$$

where $\Phi^{\left(\alpha \right)}$ is the acoustic wave function set in subdomain α, for each kind of boundary condition, and $n^{\left(\alpha \right)}$ is the corresponding boundary normal vector.

(A7) $$C_{p}^{\left(\alpha ,\beta \right)}={\int_{\Gamma_{I}}\left[\Phi^{\left(\alpha \right)}\right]}^{T}\frac{j}{\rho_{0}\omega }n^{\left(\beta \right)}\nabla \Phi^{\left(\beta \right)}+\frac{1}{Z_{n}^{*}}{\left[\Phi^{\left(\alpha \right)}\right]}^{T}\Phi^{\left(\beta \right)}ds$$

In Equation (23):

(A8) $$C_{sp}^{\left(\alpha ,s\right)}=-j\omega {\int_{\Gamma_{z}}\left[\Phi^{\left(\alpha \right)}\right]}^{T}n_{s}n^{\left(\alpha \right)}\Psi_{b}^{\left(s\right)}ds$$

where $n_{s}$ is the normal vector of plate, and $\Psi_{b}^{\left(s\right)}$ is the structural bending wave function set.

In Equation (25):

(A9) $$f_{p}{}^{\left(\alpha ,\alpha \right)}=f_{v}^{\left(\alpha ,\alpha \right)}+f_{p}^{\left(\alpha ,\alpha \right)}+f_{Z}^{\left(\alpha ,\alpha \right)}+f_{s}^{\left(\alpha ,\alpha \right)}+f_{I}^{\left(\alpha ,\alpha \right)}$$

(A10) $$f_{v}^{\left(\alpha ,\alpha \right)}=\int_{\Gamma_{v}}-\frac{j}{\rho_{0}\omega }{\left[\Phi^{\left(\alpha \right)}\right]}^{T}n^{\left(\alpha \right)}\nabla {\tilde{p}}_{q}{}^{\left(\alpha \right)}+{\left[\Phi^{\left(\alpha \right)}\right]}^{T}v_{n}^{*}ds$$

(A11) $$f_{p}^{\left(\alpha ,\alpha \right)}=\int_{\Gamma_{p}}\frac{j}{\rho_{0}\omega }n^{\left(\alpha \right)}\left(\nabla {\left[\Phi^{\left(\alpha \right)}\right]}^{T}\right)\left({\tilde{p}}_{q}{}^{\left(\alpha \right)}-p^{*}\right)ds$$

(A12) $$f_{Z}^{\left(\alpha ,\alpha \right)}=\int_{\Gamma_{Z}}-\frac{j}{\rho_{0}\omega }{\left[\Phi^{\left(\alpha \right)}\right]}^{T}n^{\left(\alpha \right)}\nabla {\tilde{p}}_{q}{}^{\left(\alpha \right)}+{\left[\Phi^{\left(\alpha \right)}\right]}^{T}{\tilde{p}}_{q}{}^{\left(\alpha \right)}ds$$

(A13) $$f_{s}^{\left(\alpha ,\alpha \right)}=\sum_{s=1}^{N_{s}}{\int_{\Gamma_{s}}\left[\Phi^{\left(\alpha \right)}\right]}^{T}\left(j\omega \cdot n_{s}n^{\left(\alpha \right)}\left({\tilde{w}}_{q}^{\left(s\right)}+{\tilde{w}}_{F}^{\left(s\right)}\right)-\frac{j}{\rho_{0}\omega }n^{\left(\alpha \right)}\nabla {\tilde{p}}_{q}{}^{\left(\alpha \right)}\right)ds$$

(A14) $$f_{I}^{\left(\alpha ,\alpha \right)}={\int_{\Gamma_{I}}\left[\Phi^{\left(\alpha \right)}\right]}^{T}\left(-\frac{j}{\rho_{0}\omega }n^{\left(\alpha \right)}\nabla {\tilde{p}}_{q}{}^{\left(\alpha \right)}+\frac{1}{Z_{\mathrm{int}}}{\tilde{p}}_{q}{}^{\left(\alpha \right)}\right)ds$$

For any two adjacent sub-domains:

(A15) $$f^{\left(\alpha ,\beta \right)}=f_{I}^{\left(\alpha ,\beta \right)}={\int_{\Gamma_{I}}\left[\Phi^{\left(\alpha \right)}\right]}^{T}\left(-\frac{j}{\rho_{0}\omega }n^{\left(\beta \right)}\nabla {\tilde{p}}_{q}{}^{\left(\beta \right)}-\frac{1}{Z_{\mathrm{int}}}{\tilde{p}}_{q}{}^{\left(\beta \right)}\right)ds$$
